# Assembling and dietary application of a local trnL metabarcoding database for *Cervusnipponkopschi* in Taohongling Nature Reserve

**DOI:** 10.3897/BDJ.12.e139269

**Published:** 2024-11-21

**Authors:** Yuqin Liu, Dandan Wang, Zhiming Cao, Wuhua Liu, Zechun Bao, Weiwei Zhang, Yongtao Xu

**Affiliations:** 1 Jiangxi Provincial Key Laboratory for Conservation Biology, Jiangxi Agricultural University, Nanchang, China Jiangxi Provincial Key Laboratory for Conservation Biology, Jiangxi Agricultural University Nanchang China; 2 Taohongling Sika Deer National Nature Reserve Administration, Pengze, China Taohongling Sika Deer National Nature Reserve Administration Pengze China; 3 Jiangxi Lushan National Nature Reserve Administration, Jiujiang, China Jiangxi Lushan National Nature Reserve Administration Jiujiang China

**Keywords:** local database, *Cervusnipponkopschi*, diet, trnL metabarcoding, high-throughput sequencing

## Abstract

The quality and completeness of the reference database have a direct impact on the accuracy of forage plant identification, thereby influencing the level of conservation and management of wildlife resources. In our research, target amplification was subjected to first-generation sequencing to assemble a local reference database using chloroplast trnL metabarcoding. We found that the primers c-h outperformed g-h as a universal DNA metabarcoding and 162 valid choloroplast trnL sequences were submitted (GenBank ID: PP081756 - PP081917), which exhibited an obvious preference for A and T nucleotides (60.49%). The haplotype diversity (*Hd*), nucleotide diversity (*Pi*) and average number of nucleotide differences (*K*) of these trnL sequences were 0.978, 0.0484 and 4.743, respectively. To assess the availability of the local database in identifying the diet of South China sika deer (*Cervusnipponkopschi*), high-throughput metabarcoding sequencing and BLAST analysis were performed. Ultimately, 25 forage plant species were identified, belonging to 19 families and 25 genera. Shrubs and herbaceous plants, such as *Potentillafreyniana*, *Persicariaperfoliata*, *Rosalaevigata* and *Ardisiajaponica* etc, dominated the forage plants. This study established a local trnL reference database that holds immense value for the forage plant identification and nutritional evaluation for sika deer and other sympatric herbivores, as well as the conservation and management of biodiversity.

## Introduction

Food could provide the necessary nutrition and energy for the life activities of a species, it is a crucial resource for sustaining the survival and growth of populations and a key area of scientific research in zooecology. In recent years, the fragmentation and loss of animal habitats have resulted from global climate change, environmental pollution and excessive exploitation and the availability of food resources is in decline. Large herbivores have faced a population decline and consistently have the highest proportions of threatened species at multiple spatial and temporal scales (Atwood et al. 2020). The South China sika deer, scientifically known as *Cervusnipponkopschi*, a large ruminant, is classified as a herbivorous mammal belonging to the order Cetartiodactyla, family Cervidae and genus *Cervus*. This is an endemic species to the East Asian monsoon region and has been listed as an endangered species by the International Union for Conservation of Nature (IUCN) in 1986 and 1988 (Blouch et al. 1998, Wei et al. 2006). Although sika deer have been classified as a Least Concerned species in 2015 by the IUCN (Harris 2015), the South China subspecies still is a national Class I protected animal in China, because of the small and isolated distribution, as well as a low inter-population genetic exchange (Zhang et al. 2016).

The Taohongling Sika Deer National Nature Reserve (hereafter, TNNR) is the predominant habitat for the South China sika deer. Throughout the years, the reserve has diligently pursued a rigorous programme of forest conservation and restoration. The vegetation succession of the Reserve has restored the plants in many zones to evergreen broad-leaved mixed forests (Jiang et al. 2012), which has also resulted in a significant reduction in understorey forage resources (i.e. shrubs and herbs) and a limitation of the environmental carrying capacity. As a result, sika deer frequently forage beyond the Reserve boundary that poses challenges to wildlife conservation and management of the Reserve. Dietary analysis plays an instrumental role in providing essential theoretical foundations for the conservation of endangered wildlife and other studies (Hoenig et al. 2021). A diet ecology study of wildlife can better acquire feeding behaviour, food selection, food resource utilisation efficiency and intricate food webs. Thus, acquiring precise data regarding animal diets is of utmost importance for animal development, reproduction, management and even local plant community diversity and dynamics (Zheng and Bao 2004, Nielsen et al. 2017).

The traditional dietary analysis includes field observations, stomach content analysis (Bai et al. 2022), faecal microhistological analysis etc. (Zhu et al. 2019), which have been employed for the yellow-throated marten (Choi et al. 2015, Zhu et al. 2019, Zhu et al. 2019), Alpine musk deer (Xu et al. 2018) and Tibetan antelopes (Cao et al. 2008). Sika deer, as herbivorous ruminants, have long food retention times in the digestive system and this imposes certain limitations on the use of traditional analysis. Subsequently, Wei (2022) emphasised that non-invasive sampling mitigated the challenges that endangered wildlife studies faced, particularly in conservation biology fields utilising faecal samples. With the continuous advancement of high-throughput sequencing, DNA metabarcoding techniques in identifying the diet composition of herbivorous animals have been utilised efficiently, based on obtained operational taxonomic units (OTUs) (Kartzinel et al. 2015), tracing the composition of foraged plant species in the faecal DNA. The DNA barcoding approach utilises a standardised, relatively short DNA fragment within the genome as a molecular marker and identification or variant classification relies on nucleotide sequences differences (Guo et al. 2021, Wang and Duan 2022, Santos-Perdomo et al. 2024). It has addressed the bottlenecks of traditional dietary exploring, offering non-invasiveness and efficient species identification, eliminating subjective interpretation and rapidly analysing large-scale animal and plant samples (Taberlet et al. 2012, Zhang et al. 2022).

DNA metabarcoding has the potential to qualitatively and quantitatively analyse the composition of herbivore diets (Guo et al. 2021). In metabarcoding analyses, the taxonomic assignment is crucial to place sequencing data in biological and ecological contexts (Mugnai et al. 2023), which is typically achieved by matching DNA sequences to the reference library (a database of DNA sequences) (Edgar 2018). However, it is estimated that less than 20% of plants have been represented in public databases (Hebert et al. 2016). The problem of low species level identification rates by DNA barcoding in plants is exacerbated by the fact that reference databases are far from being comprehensive (Kolter and Gemeinholzer 2021). The species absent and erroneous identification are likely to produce wrong taxonomic assignments of query barcodes (Bush et al. 2019, Paz and Rinkevich 2021). Restricting the reference databases to species present in a specific research region may be a good direction. Application effectiveness of DNA metabarcoding can be realised with the construction of specific local reference databases (Mugnai et al. 2023). Soininen et al. (2009) established a database comprising 842 plant species in the Arctic Region, which significantly improved the success rate of diet identification between *Microtusoeconomus* and *Myodesrufocanus*, providing a more detailed and objective understanding of diet composition. Moreover, the prior study also emphasised that it is necessary to establish specific regional- even national-level biological barcode databases applying to *Rusaunicolor* diet (Zhang et al. 2020) and the biodiversity analysis of zooplankton (Gao et al. 2020), thus reducing the impact of sequence matching errors before bioinformatic annotation.

The accuracy and precision of dietary research have a direct impact on the level of wildlife resource management (Lu et al. 2020). For a cleaner and more accurate taxonomic identification of the diet composition of sika deer as well as other sympatric herbivores in the TNNR, this study attempts to assemble a local reference database of potential forage plants chloroplast trnL (UAA) gene and provide a source of sequence alignment for diet analysis. Further application of dietary analysis can significantly enhance our understanding of resource utilisation patterns, trophic structures and interspecific interactions amongst South China sika deer and their sympatric herbivores.

## Material and methods

**Study areas**

The TNNR is located on the south bank of the middle-lower reaches of the Yangtze River, along the northern border of the subtropical zone, Pengze, Jiangxi Province, China. The Reserve represents the largest distribution area for the South China sika deer and is a natural habitat for numerous wildlife species. The total area of TNNR is 12,500 hm^2^, the core area is 2,670 hm^2^, the experimental area is 1,830 hm^2^ and the buffer zone is 8,000 hm^2^. The core zone is for conservation and most of the sika deer live in this area; the experimental zone is for human activities and regulated development; a buffer zone has some allowable human activities, thereby mitigating artificial interference for the core zone (Liu et al. 2008). The TNNR experiences a subtropical monsoon climate and is predominantly composed of low mountains and hills, with elevations ranging from 100 to 500 m above sea level (Wang et al. 2021). The zonal vegetation primarily consists of broad-leaved evergreen forests, which also include coniferous, mixed coniferous, broad-leaved and bamboo forests, amongst others.

**Sample collection**

**Figure 1.**
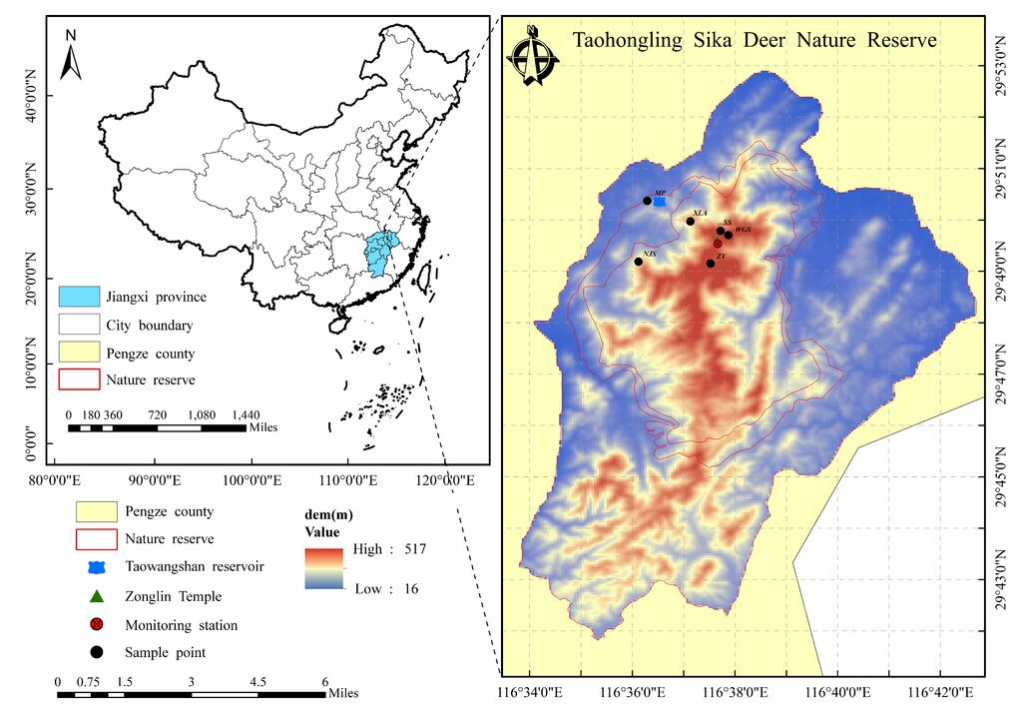
Sample sites at the Taohongling Sika Deer Nature Reserve (MP: Nursery bases; SS: Fir forests; NJS: NieJiashan; XLA: XianLingAn; WGS: WuGuiShi; ZY: Bamboo garden).

Six sampling sites, including Nursery bases, XianLingAn, fir forests, NieJiashan, WuGuiShi and the Bamboo Garden, were designated in the frequent activity areas of sika deer at the TNNR in the autumn of 2022 (Fig. 1). Using transect and plot surveys to identify the potential forages of sika deer, three to five transects (2 km surveyed per transect) were set up at each sampling site and each transect was randomly positioned in the study area, covering different habitat types. During the collection of plant samples, clean gloves were worn to prevent contamination of the samples and accidental contact with toxic plants. Some 1-3 fresh leaves were gathered and preserved using silica gel for drying and sealed for each plant species, ensuring the subsequent research.

**DNA extraction and PCR amplification**

To avoid impacting the yield and concentration of DNA during extraction, plant samples were thoroughly dried using silica gel before the experiment. After completely drying, 20 mg (not exceeding 30 mg) of dried leaf tissue were placed in a 2 ml EP tube with one steel bead. The plant tissue was then ground for 80 seconds at a frequency of 45 Hz using a plant tissue homogeniser. Plant samples that were not successfully homogenised were subjected to repeated grinding until they became a fine powder. To prevent DNA degradation, the ground plant tissue was transferred promptly. In this study, the DNA extraction of the samples was performed using the Fore Gene Plant DNA Isolation Kit (Chengdu). The DNA Optical Density (OD) value was measured by an ultraviolet spectrophotometer and the A260/A280 of most DNA extracts was between 1.70 and 2.21, indicating highly purified DNA. Then, the DNA was stored in a -20℃ freezer until further use.

**Figure 2.**
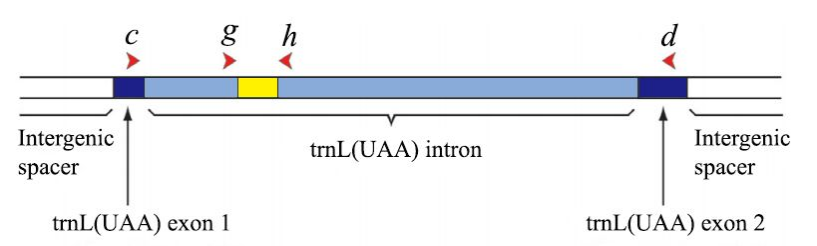
Structural positions of the primers c-h and g-h in the chloroplast trnL (UAA) gene.

The sequences for the trnL (UAA) gene universal primers, designated as g (5’-GGGCAAT CCTGAGCCAA-3’) and h (5’-CCATTGAGTCTCTGCACCTATC-3’) and the primers c (5’-CGAAATCGGTAGACGCTACG-3’) and h (5’-CCATTGAGTCTCTG CACCTATC-3’) were employed to amplify plant DNA (Taberlet et al. 2007). The primer sequence positions within the trnL (UAA) gene structure are illustrated (Fig. 2). The PCR amplifications were carried out for primers c-h in a total volume of 25 μl containing 12.5 μl PCR mix, 1 μl DNA template, 1 μl each of forward and reverse primers and 9.5 μl ddH_2_O and a PCR negative control (sterile water) was added. The reaction conditions were as follows: denaturation at 94°C for 4 min, followed by 35 cycles at 94°C for 30 s, 56°C for 30 s and 72°C for 45 s, with a final step of 10 min at 72°C. The PCR amplification system for primers g-h was prepared in a 25 μl reaction volume consisting of 12.5 μl PCR mix, 2.5 μl DNA template, 0.8 μl of each forward and reverse primer and 8.4 μl ddH_2_O. The reaction conditions were as follows: denaturation at 95°C for 3 min, followed by 35 cycles at 95°C for 30 s, 56°C for 30 s and 72°C for 45 s, with a final step of 10 min at 72°C. PCR products were subjected to quality evaluation using 1% agarose gel electrophoresis (200 V, 100 mA, 30 min). The bands with clear and qualified products were selected for further analysis and sent to Shanghai Saiheng Biological Technology Co., Ltd. for DNA fragment single pass sequencing. The raw sequencing data were saved in FASTQ format.

**Data s tatistics**

The obtained sequences were aligned using Clustal W and trimmed. Afterwards, MEGA-11 software was employed to calculate the base composition and variation information of aligned sequences. The analysis includes conserved sites, variable sites, parsimony-informative sites, sequence length, base composition and the value for transitions and transversions. The phylogenetic tree was constructed using the Maximum Likelihood (ML) method based on the Tamura 3-parameter mode. The system utilised bootstrap analysis with 1000 iterations to assess the confidence of the tree nodes. Additionally, DnaSP was employed to calculate the total number of haplotypes for each population and the overall number of haplotypes across all species, haplotype diversity (*Hd*), nucleotide diversity (*Pi*) and average number of nucleotides (*K*). *Hd* and *Pi* values are two crucial metrics for assessing the level of variation within a population. A higher value indicates a greater degree of polymorphism within the population.

**Diet a pplication**

To validate the application and effectiveness of DNA sequences in the plant reference database, 15 fresh faecal samples of sika deer were collected from TNNR in summer. Two faecal pellets were randomly taken from each faecal sample and mixed to form a single composite sample with three repetitions, a total of five mixed samples were prepared. Total DNA was extracted by the protocol for high-throughput sequencing and sent to Shanghai Personal Biotechnology Co., Ltd. for sequencing. Purified amplicons were pooled in equimolar and paired-end sequenced (2 × 300) on an Illumina MiSeq platform (Illumina, San Diego, USA). Demultiplexed sequences from each sample were quality filtered and trimmed, denoised and merged and then the chimeric sequences were identified and removed using the QIIME2 dada2 plugin to obtain the feature table of OTUs (Wang et al. 2023). All OTUs were assigned to taxonomic units by referring to the local database using the NCBI-hosted BLAST web server, which provides (sufficiently) quick search results (Cock et al. 2015). Then sequences were input in the “BLASTn” suite, with thresholds of identity > 97% and e-value < 1.0 e^-50^. If an OTU matched two or more taxa, it was assigned to a higher taxonomic level that included all taxa (Hou et al. 2021).

## Results

**DNA extraction from plant samples**

A total of 290 plant samples were collected in this study from TNNR, encompassing 282 species from 90 families and 204 genera, with eight species being duplicates (Suppl. material 1). Plant DNA was extracted from green, dried leaf samples, with a total of 268 plant DNA samples and a yield rate of 94.4%. These findings demonstrate the broad applicability of plant DNA extraction kit used in our research. For individual samples that showed no bands or unclear bands, three replicates of the experiments were conducted. A total of 17 plant samples belonging to 12 different families, i.e. Asteraceae, Rosaceae, and Rutaceae, did not exhibit distinct and bright bands in the gel electrophoresis analysis.

**Amplification and sequence analysis**

**Figure 3.**
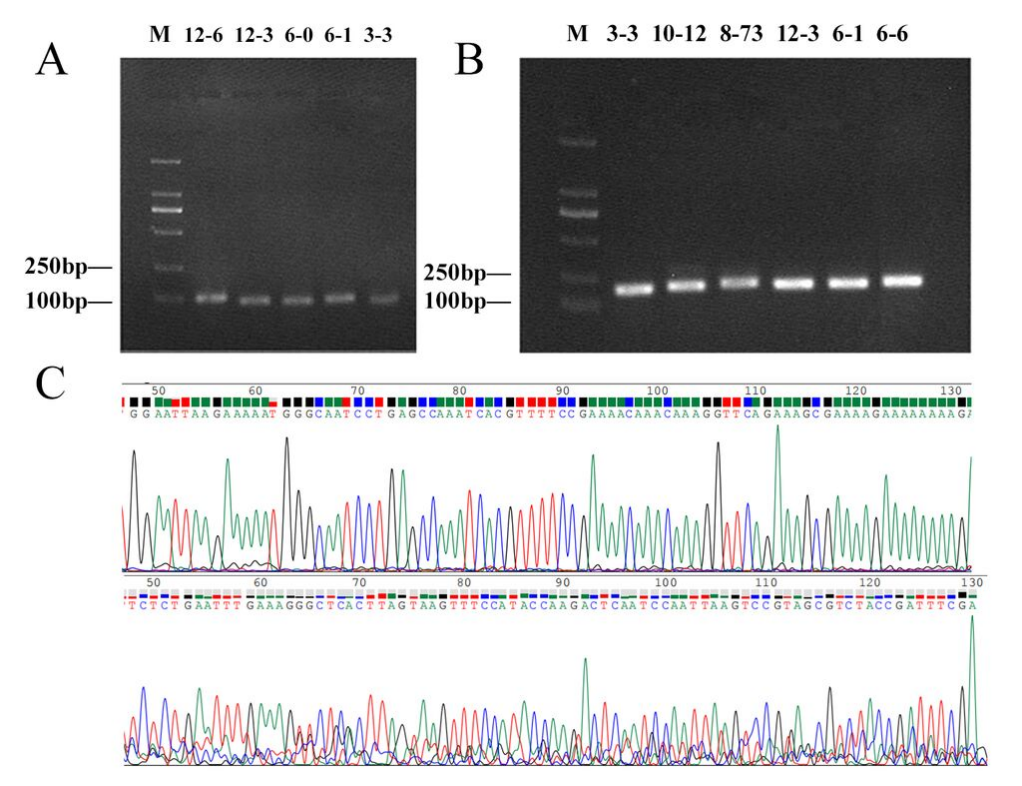
Gel electrophoresis test and sequence peak maps. **A** Amplification detection of primers g-h, length approximately 50 bp; **B** Amplification detection of primers c-h, length approximately 150 bp (M: DL2000 DNA Marker, the sample numbers above the lanes followed by Clematispuberulavar.ganpiniana, *Lactucasativa*, *Castanopsistibetana*, *Cunninghamialanceolata*, *Rorippacantoniensis*, *Hedyotischrysotricha* and *Lonicerajaponica* from left to right); **C** Normal sequence peaks (*Artemisiacaruifolia*) and cross-peaks (*Dicranopterispedata*).

**Figure 4.**
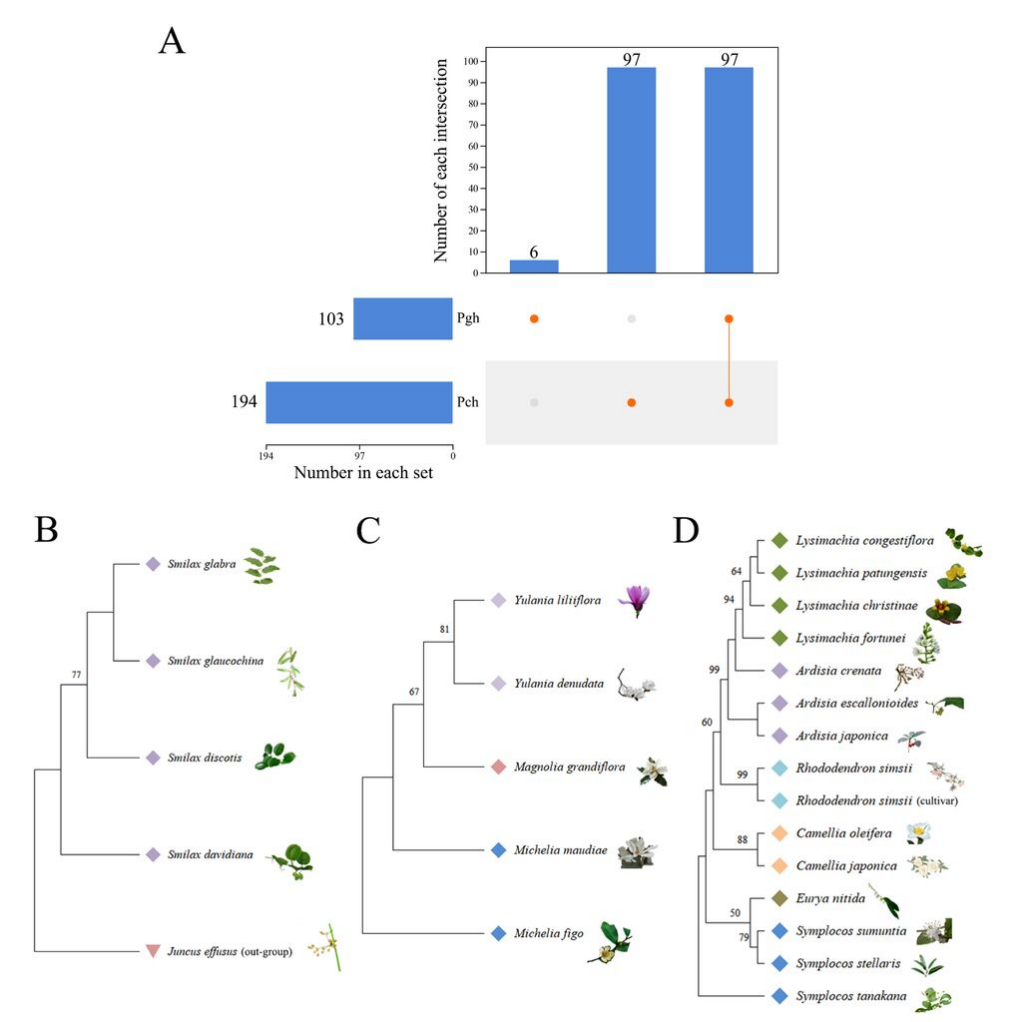
Venn analysis and Phylogenetic tree maps at the order level. **A** Single dot indicates the number of endemic species identified within a group and multiple dots connected to the line indicate the number of shared species between groups; **B** Phylogenetic tree map for the order Liliales (including an outgroup); **C** Phylogenetic tree map for the order Ericales; **D** Phylogenetic tree map for the order Magnoliales. The numbers associated with each branch represent the bootstrap values obtained from the Maximum Likelihood analysis (note: the same colour represents the same genus).

In this study, a total of 268 DNA samples (including three replicates) were subjected to PCR amplification and gel electrophoresis analysis, the part of gel electrophoresis bands shown in Fig. 3A and 3B. Results indicate that primers c-h amplified the target DNA sequences in 194 plant samples successfully, representing 146 genera and 76 families, including Poaceae, Rosaceae and Lamiaceae. On the other hand, a total of 103 plant samples were amplified using primers g-h, belonging to 85 genera in 48 families. The number of common species was 97 and the specific species was only six in primers g-h (Fig. 4A). Amplification success rate for primers g-h was 39.25%, whereas for primers c-h, it was 74.34%, the results demonstrating that primers c-h outperform g-h in the amplification of plant DNA sequences. In total, 202 PCR products were submitted for sequencing, amongst them, 16 samples did not meet the sequencing requirements and 24 samples exhibited polymer structure and cross-peak phenomena (Fig. 3C) and these samples were excluded from subsequent analysis. Ultimately, 162 high-quality sequences were successfully obtained and submitted to GenBank using bankit (GenBank ID: PP081756 - PP081917) and compiled into the local reference database of potential diets for sika deer. The length of these sequences ranged from 162-255 bp, see Suppl. material 2.

The 162 sequences revealed an average base composition of 37.44% for T (thymine), 22.35% for C (cytosine), 23.05% for A (adenine) and 17.16% for G (guanine). Notably, there was a significantly higher content of A+T (accounting for 60.49%), indicating a significant preference for A and T in the base composition. The average transition/transversion ratio (*Ts/Tv*), represented by the value R, was found to be 1.0. Based on 162 sequences, 70 polymorphic sites, 42 singleton sites and 28 parsimony-informative sites were obtained. A total of 71 haplotypes were detected, the average number of nucleotide differences per site (*K*) was calculated to be 4.743, haplotype diversity was found to be 0.978 and nucleotide diversity (*Pi*) was 0.04840. At the order level, the 34 orders exhibited haplotype diversity values ranging from 0.50000 to 1.00000, while the nucleotide diversity values ranged from 0.00518 to 0.22655. The results indicated the nucleotide diversity of Magnoliales and Liliales was relatively high, with *Pi* values exceeding 0.1 and exhibited a wide range of sequence variations and significant nucleotide sequence differences between individuals; Lilium and other orders occupied relatively lower nucleotide diversity, with higher sequence similarity amongst individuals. The order Magnoliales exhibited the highest number of polymorphic sites and had larger nucleotide diversity values correspondingly (Table 1, Fig. 4). The phylogenetic tree, specifically Liliales, Ericales and Magnoliales, revealed that taxa belonging to the same genus cluster together, indicating closer sequence relationships amongst them and the database of this research had reliability (Fig. 4B, C and D).

**Sequence alignment and diet identification**

**Figure 5.**
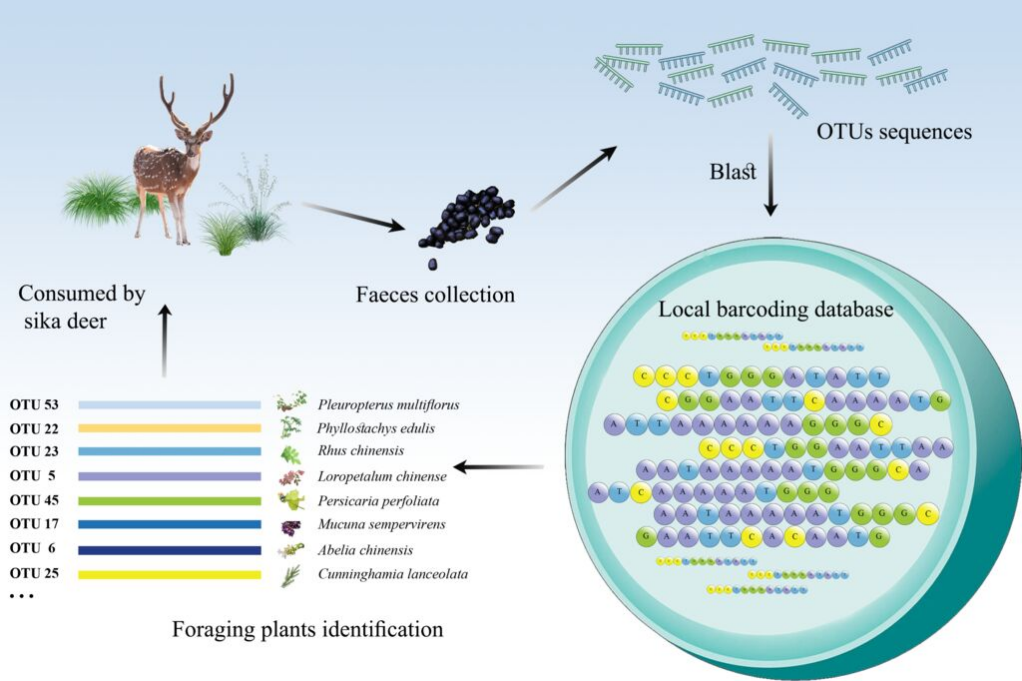
Foraging plants identification of sika deer, based on the local barcoding databases, and the "..." means other OTUs.

Following strict data quality control, multiple sequences with 100% similarity were clustered into one OTU to reduce the computational burden of subsequent species annotation. Finally, 56 OTU sequences were selected to align with the reference database and 25 chloroplast trnL sequences were matched successfully to local reference database, based on the BLAST platform. These sequences were further analysed and classified, belonging to 19 families and 25 genera (Fig. 5 and Table 2). In total, eight herbaceous plants included *Phyllostachysedulis*, *Potentillafreyniana*, *Persicariaperfoliata* and *Pleuropterusmultiflorus*; six shrubs, such as *Viburnumdilatatum*, *Rosalaevigata*, *Ardisiajaponica* and *Akebiatrifoliata*; eight trees, i.e. *Cunninghamialanceolata*, *Camphoraofficinarum* and *Callicarpabodinieri*; and three climbers, such as *Kadsuralongipedunculata*, *Mucunasempervirens* and *Trachelospermumjasminoides* were identified. The 25 forage plants identified in this study were mainly herbs and trees, with most possessing medicinal value. These findings provide preliminary evidence to confirm the diet of sika deer at the species level.

## Discussion

**Sample quality and barcode selection**

The quality and selection of plant samples play a crucial role in efficient database construction. Most plants begin to germinate during the warm spring and enter a period of colour change and leaf shedding subsequently, then complete their life cycle in winter (Barichivich et al. 2013). Fresh leaves or seedlings in the current year can maintain the DNA structural integrity and physiological activity of the plants (Gao et al. 2012). The fresh leaves contribute to the DNA extraction during the DNA extraction process.

Selecting the optimal DNA barcode should be crucial considering the inter- or intraspecific characteristics of different plant taxa (Erickson et al. 2008). The mitochondrial COI gene (~ 650 bp) exhibits considerable interspecific variability and is highly effective in animal identification (Luo et al. 2011). However, the lower variation and slower evolutionary rate of the COI gene are not applicable for plant identification (Guo et al. 2021). Currently, the selection of DNA barcodes for higher plants primarily focuses on nuclear and chloroplast genes. However, the vast number and complex ploidy of plant species, frequent inter-specific hybridisation, as well as the variation in evolutionary rates amongst different genes, contribute to the complexity of plant genetic evolution. Mallott et al. (2018) indicated that the trnL gene can be utilised for quantitatively assessing dietary differences between intra- and interspecific with a more powerful resolution. P6 loop region (10-143 bp) of the trnL gene, located within the intron sequence of the chloroplast gene trnL (UAA), can be well suited for the identification of plants in faecal samples (Taberlet et al. 2007). The chloroplast trnL intron was selected as a barcode due to its high taxonomic coverage and resolution (Hou et al. 2021).

In this study, a comparative analysis between the primers c-h and g-h amplification was performed and the primer c-h indicated better amplification efficiency, but partial plant species showed primers g-h preference. Additionally, it is speculated that identifying all the plant species is difficult because of the loss of universality and general applicability primers, which may be one of the factors influencing the accuracy of our species identification rates. Although many studies have searched for a universal plant barcode, none of the available loci works across all species (Chen et al. 2010). Some taxonomists have suggested that multi-locus may be necessary to discriminate plant species (Hebert et al. 2004). Hollingsworth et al. (2009) compared the performance of seven leading candidate DNA regions (atpF-atpH spacer, matK gene, rbcL gene, rpoB gene, rpoC1 gene, psbK-psbI spacer and trnH-psbA spacer) and recommended the 2-locus combination of rbcL+matK as the optimal plant compound barcodes. However, it was also demonstrated that there was no clear improvement of discriminatory ability on the species level by using multiple loci. Frequent hybridisation and polyploidisation complicate species-level identification in plants and it is proving difficult to establish both single and combined barcode markers that offer adequate resolution at the species level (Kolter and Gemeinholzer 2021). A specific barcode is a fragment of DNA sequence that has a sufficiently high mutation rate to enable species identification within a given taxonomic group (Li et al. 2014). In the future, specific barcodes may provide new perspectives in the search for rapid and accurate methods for species discrimination, especially for closely-related plants.

**Local barcoding database of potential foraging plants**

The accuracy of species identification was determined by the quality and completeness of reference databases (Yang 2017). It is still difficult to identify the species level of plants precisely in specific regions, given the genetic variations resulting from different geographical distribution of plants. Public reference databases contain available sequence information assembled from around the world. If the generalist database were almost complete or had great coverage of the target taxa in the geographical region under study, it may be an evident solution for taxonomic assignment (Mugnai et al. 2023). However, although this is an ideal situation, the sequences in reference databases belonging to taxonomic groups out of the studied region might lead to false assignments. Therefore, it may be insufficient to perform diet identification, nutritional analysis and habitat restoration in specific regions based only on public reference databases (Pompanon et al. 2012, Cheng and Zhang 2022. The local databases are tailored to a specific geographical region, which has the potential to increase species-level barcoding success by eliminating congeners that have no natural occurrences in the study region (Kolter and Gemeinholzer 2021). In this research, a total of 25 forage plant species were obtained by OTU alignment, lots of plants being the same as the forages consumed by sika deer in winter (*Cunninghamialanceolata*, *Akebiatrifoliata* etc.) (Wang et al. 2023). The assembly of a local plant barcoding database has proven to be effective and relatively specific for the diet consumed by sika deer at the species level in this study. Together with the less human subjective factors make it viable to carry out the diet identification and nutritional analysis for sika deer and other herbivores in TNNR. The unaligned sequences were likely attributed to the incomplete species inventories, which do not encompass the entire diet of sika deer, consequently affecting the species identification rate. However, we still need to continuously sample new specimens for individual identification and sequencing obtained explicitly from the study region, to enrich the local database and eventually cover the whole biodiversity. Thus, it is essential to establish a reliable local plant barcoding database through a comprehensive background investigation to avoid insufficient sequence representation (Deagle et al. 2010, Liu et al. 2018, Shao et al. 2019).

**Diet composition**

Diet composition analysis contributes to assessing the nutritional intake of the wildlife. Shrubs, with more protein and mineral elements than herbs, occupy a vital proportion of the forage plants for sika deer. In the winter when food resources may be scarce, sika deer prefer woody vegetation, particularly shrubs, ensuring a balanced diet (Zhong 2021). It was amazing to find that Chinese firs, with hard thorns, poor palatability and high content of tannins, were consumed by sika deer (Fedorov and Ryazanova 2021). The reason for this was that large amounts of artificial forest were planted to respond to the national Grain-for-green policy in the 1960s. Coniferous plants (i.e. Chinese fir and Masson pine) were widely cultivated because of their strong environmental adaptability. A large number of fir forests have been rigorously preserved since the establishment of the provincial nature reserve in 1981. Long-term survival adaptation strengthens the selection of Chinese fir resources for sika deer and other sympatric herbivores.

Different herbivores have different tolerances to tannins, with ruminants having been shown to tolerate a certain amount of tannins in their natural diet (Xing et al. 2022). Selection and use of plants for red deer (*Cervuselaphus*) were partly associated with high levels of active tannins (Zweifel‐Schielly et al. 2011). Preference trials with captive roe deer (*Capreoluscapreolus*) have suggested an active selection for a low dose of hydrolysable tannins (Clauss et al. 2003). Several farmers have discovered that tannins augment the reproductive and survival rates of fawns in captivity (Xing et al. 2022). To some extent, this may also explain the cause of consuming fir trees for sika deer. From our database, many types of forage plants belonging to Chinese herbal medicine have been identified, for instance, *Pleuropterusmultiflorus*, *Persicariaperfoliata*, *Kadsuralongipedunculata*, *Ardisiajaponica*, *Loropetalumchinense* etc. Sika deer are also famous for their economic and medical value in traditional Chinese medicine, such as velvet antlers, blood and skin (Guan et al. 2017). The intricate mechanisms between the ingestion of Chinese herbal medicines and the metabolic transformations of sika deer need further exploration.

## Conclusions

This study provided new insight into diet identification based on high-throughput sequencing and a local database for sika deer and other sympatric herbivores, which are essential for clarifying the dietary nutrition, food utilisation and transmission and the structure and functionality of ecosystems. However, the construction of a DNA barcoding database is a complex and long-term process, requiring continuous optimisation and improvement. In the follow-up study, a complete and local DNA barcoding database needs to be constructed to cover more potential forage plants and to further improve the accuracy of diet identification for the herbivores.

## Supplementary Material

21710503-C048-5867-AD1A-B98E8E6F78C5 10.3897/BDJ.12.e139269.suppl1 Supplementary material 1 Collection information Data type Table Brief description Collection information of potential forage plants for sika deer in TNNR. File: oo_1173673.docx https://binary.pensoft.net/file/1173673 Yuqin Liu

AAD3DA50-CFBF-5286-B1F3-B4D2717CD549 10.3897/BDJ.12.e139269.suppl2 Supplementary material 2 Local reference database Data type Table Brief description The local reference database based on the trnL gene (containing 162 plants’ DNA sequence information). File: oo_1173674.docx https://binary.pensoft.net/file/1173674 Yuqin Liu

## Figures and Tables

**Table 1.** Genetic diversity parameters of 162 sequences (including variable sites, sample size, number of haplotypes, haplotype diversity, nucleotide diversity and the number of average nucleotide differences)

**Orders**	**Variable site**	**Sample size**	**Number of haplotype (Nh)**	**Haplotype diversity (Hd)**	**Nucleotide diversity (Pi)**	**Number of average nucleotide difference(K)**
Liliales	2	4	2	0.500	0.005	1.000
Cupressales	5	2	2	1.000	0.027	5.000
Dipsacales	12	5	5	1.000	0.038	6.500
Lamiales	18	15	11	0.933	0.026	4.152
Fabales	35	6	6	1.000	0.080	13.667
Ericales	43	15	9	0.924	0.070	12.390
Poales	30	18	11	0.915	0.058	9.935
Malpighiales	16	5	5	1.000	0.042	7.800
Malvales	6	2	2	1.000	0.029	6.000
Asterales	5	4	3	0.833	0.013	2.500
Fagales	16	3	2	0.667	0.055	10.667
Gentianales	37	7	7	1.000	0.082	6.000
Ranunculales	25	7	6	0.952	0.069	10.952
Magnoliales	99	5	4	0.900	0.227	14.762
Rosales	31	19	11	0.936	0.041	7.550
Solanales	2	3	2	0.667	0.007	1.333
Caryophyllales	27	9	9	1.000	0.068	11.250
Dioscoreales	4	2	2	1.000	0.018	4.000
Asparagales	32	3	3	1.000	0.119	22.333
Sapindales	24	6	4	0.800	0.060	11.200

**Table 2.** The 25 forages that were successfully identified based on the local reference database for sika deer

**Number**	**Forage plants**	**Sequence information**	**Sequence length(bp)**
OTU2	*Dalbergiahupeana*	GACTTAATTGGATTGAGCCTTGGTATGGAAACGTACCAAGTGATAACTTTCAAATTCAG AGAAACCCCGGAATTAACAATGGGCAATCCTGAGCCAAATCCCGTTTTCTGAAAGCAAA GAAAAATTAAGAAAGAAAAAGG	140
OTU4	*Kadsuralongipedunculata*	GACTTGATTGGATTGAGCCTTAGTATGGAAACCTACTAAGTGGTAGCTTCCAAATTCAG AGAAACCCTGGAATTAAAAATGGGTAATCCTGAGCCAAATCCTGTTTTCAGAAAACAAT GGTTTAGAAGTTTAGAAAGCGAGAATAAAAAAAAGGTAGG	158
OTU5	*Loropetalumchinense*	GACTTGATTAGCTTGAGCCTTGGTATGGAAACCTGCTAAGTGGTAACTTCCAAATTCAG AGAAACCCCGGAATTCAAAATGGGCAATCCTGAGCCAAATCCTGTTTTCCGAAAACAAA GACAAGGGTTCAGAAAGCGAGAATCAAAATAAAAAAAG	156
OTU6	*Abeliachinensis*	GACTTAATTGGATTGAGCCTTGGTATGGAAACCTACTAAGTGATAACTTTCAAATTCAG AGAAACCCTGGAATTAATAAAAATGGGCAATCCTGAGCCAAATCCAGTTTTACGAAAAC AAGGGTTCAGAAAGCTAAAATCAAAAAG	146
OTU9	*Phyllanthusurinaria*	GACTTAATTGAATTGAGCCTTGGTATGGAAATCTACCAAGTGATAACTTTCAAATTCAG AGAAACCCTGGAATTAAAAATGGGCAATCCTGAGCCAAATCCAGTTTTCTGAAAACAA ACAAAGGTTCGTATCATAAAGATAGAATAAATAAAG	153
OTU17	*Mucunasempervirens*	GACTTAATTGGATTGAGTCTTGGTATGGAAACTTACCAAGTGAGAACTTTCAAATTCAG AGAAACCCTGGAATTCACAATGGGCAATCCTGAGCCAAATCCTCTTTTTCGAAAACAAA GATTTAAAGGAAAATAAAAAAGGG	142
OTU19	*Zeamays*	GACTTGATTGTATTGAGCCTTGGTATGGAAACCTGCTAAGTGGTAACTTCCAAATTCAG AGAAACCCTGGAATGAAAAATGGACAATCCTGAGCCAAATCCCTTTTTTGAAAAACAAG TGGTTGTCAAACTAGAACCCAAAGAAAAG	147
OTU22	*Phyllostachysedulis*	GACTTGATTGTATTGAGCCTTGGTATGGAAACCTGCTAAGTGGTAACTTCCAAATTCAG AGAAACCCTGGAATTAAAAAAGGGCAATCCTGAGCCAAATCCGTGTTTTGAGAAAACAA GTGGTTCTCGAACTAGAATCCAAAGGAAAAG	149
OTU23	*Rhuschinensis*	GACTTAATTGGATTGAGCCTTGGTATGGAAACCTACCAAGTGATAACTTTCAAATTCAG AGAAACCCTGGAATCAAAAATGGGCAATCCTGAGCCAAATCCTATTTAATGAGAACAAA AACAAACAAGGGGTCAGAACGGGAGAAAGAG	149
OTU25	*Cunninghamialanceolata*	GACTTAAATTTTTTGAGCCTTGGTATGGAAACTTACCAAGTGATAGCATCCAAATCCAG GGAACCCTGGGATATTTTGAATGGGCAATCCTGAGCCAAATCCGATTTCTGGAGACAA TAGTCTCCTATCCTAGAAAGG	138
OTU27	*Lysimachiacongestiflora*	GACTTGATTAGCTTGAGCCTTGGTATGGAAACCTGCTAAGTGGTAACTTCCAAATTCAG AGAAACCCCGGAATTCAAAATGGGCAATCCTGAGCCAAATCCTCTTTTTCGAAAACAAA GATTTAAAGGAAAATAAAAAAGGG	142
OTU28	*Viburnumdilatatum*	GACTTAATTGAATTGAGCCTTGGTATGGAAACCTACTAAGTGAGAACTTTCAAATTCAG AGAAACCCTGGAATTAATAAAAATGGGCAATCCTGAGCCAAATCCTGTTTTCCGAAAAC AAACAAAGAATCGAAAAAAAG	139
OTU29	*Rosalaevigata*	GACTTAATTGGATTGAGCCTTGGTATGGAAACCTACCAAGTGATAACTTTCAAATTCAG AGAAACCCTGGAATTAAAAATGGGCAATCCTGAGCCAAATCCCGTTTTATGAAAACAAA CAAAGTTTGCGAAAGCGAGAATAAAAAAAAG	149
OTU37	*Trachelospermumjasminoides*	GACTTAATTGGATTGAGCCTTGGTAAGGAAACCTACTAAGTGATGACTTTCAAATTCAG AGAAACCCCGGAATTAAGAAAAAGGGCAATCCTGAGCCAAATCCTATTTTCCACAAACA AAGGTTCAGAAAACGAAAACAAG	141
OTU39	*Citrusreticulata*	GACTTAATTGGATTGAGCCTTAGTATGGAAACTTACTAAGTGATAACTTTCAAATTCAG AGAAACCCAGGAATTAAAAATGGGTAATCCTGAGCCAAATCCTCTTCTCTTTTCCAAGA ACAAACAGGGGTTCAGAAAGCGAAAAAGGGG	149
OTU40	*Ardisiajaponica*	GACTTAATTGGATTGAGCCTTAGTATGGAAACCTACTAAGTGAGAACTTTCAAATTCAG AGAAACCCTGGAATTAATAAAAATGGGCAATCCTGAGCCAAATCCTCTTTTTCGAAAAC AAAGATTAAAGGAAAATAAAAAAGAGG	145
OTU42	*Camphoraofficinarum*	GACTTGGTTGGATTGAGCCTTGGTATGGAAACCTACTAAGTGATAACTTCCAAATTCAG AGAAACCCTGGAATTAAAAATGGGCAATCCTGAGCCAAATCCTGTTTTCAGAAAACAAG GGTTCAGAAAGCGAGAACCAAAAAAG	144
OTU44	*Potentillafreyniana*	GACTTAATTGGATTGAGCCTTGGTATGGAAACCTACCAAGTGATAACTTTCAAATTCAG AGAAACCCTGGAATTAAAAATGGGCAATCCTGAGCCAAATCCCGTTTTATGAAAACAAA CAAGGGTTTCATAAACCGAGAATAAAAAAG	148
OTU45	*Persicariaperfoliata*	GACTTAATTGGATTGAGCCTTGGTATGGAAACTTACTAAGTGAGAACTTTCAAATTCAG AGAAACCCTGGAAGTAAAAAAGGGCAATCCTGAGCCAACTCCTGCTTTCCAAAAGGAA AGAAAAAGAG	127
OTU48	*Euryanitida*	GACTTAATTGGATTGAGCCTTGGTATGGAAACCTACTAAGTGATAACTTTCAAATTCAG AGAAACCCTGGAATTAATAAAAATGGGCAATCCTGAGCCAAATCCTGTTTTTCGAAAAC AAACAAAGATTCAGAAAGCGAAAATCAAAAAAG	151
OTU49	*Callicarpabodinieri*	GACTTAATTGGATTGAGCCTTGGTATGGAAACCTACTAAGTGAGAACTTTCAAATTCAG AGAAACCCCGGAATTAATAAAAATGGGCAATCCTGAGCCAAATCCTGTTTTCTCAAAAC AAAGGTTCAAAAAACGAAAAAAAAG	143
OTU50	*Celtisbiondii*	GACTTAATTGGATTGAGCCTTGGTATGGAAACCTACCAAGTGATAACTTTCAAATTCAG AGAAACCCTGGAATTAAAAAAAATGGGCAATCCTGAGCCAAATCCGGTTTTCTGAAAA CAAACAAGGATTCAGGATTCAGAAAGCGATAATAAAAAAGAATCG	162
OTU53	*Pleuropterusmultiflorus*	GACTTAATTGGTTTGAGCCTTAGTATGGAAACCTACTAAGTGAGAACTTTCAAATTCAG AGAAACCCTGGAATTAAAAAAATGGGCAATCCTGAGCCAACTCCTTCTTTCCAAAAGGA AGAAAAAAG	127
OTU54	*Akebiatrifoliata*	GACTTGATTGGATTGAGCCTTGGTATGGAAACCTACTAAGTGATAACTTTCAAATTCAG AGAAACCCTGGAATGAAAAATGGGCAATCCTGAGCCAAATCCTGTTTTCAGAAAAAAAA AGGTTCAGAAAGCGAGATTAAAAAAATAAAGGAAG	153
OTU55	*Reynoutriajaponica*	GACTTAATTGGTTTGAGCCTTAGTATGGAAACCTACTAAGTGAGAACTTTCAAATTCAG AGAAACCCTGGAATTAAAAAAATGGGCAATCCTGAGCCAACTCCTGCTTTCCAAAAGG AAAGAAAAAGAG	129
