# Supplementary material for: Assembling and dietary application of a local trnL metabarcoding database for Cervusnipponkopschi in Taohongling Nature Reserve
Source: Biodivers Data J. 2024 Nov 21;12:e139269. doi: 10.3897/BDJ.12.e139269 (PMC11605298; doi:10.3897/BDJ.12.e139269)
Supplement: Supplementary material 1 — Collection information [file bdj-12-e139269-s001.docx]

**Supplementary material table S1.** Collection information of potential forage plants for sika deer in TNNR

| **Number** | **Name** | **Potential forage plants** | **Order** | **Family** | **Genus** |
| --- | --- | --- | --- | --- | --- |
| 1 | 1-1 | *Lespedeza thunbergii* | Fabales | Fabaceae | *Lespedeza* |
| 2 | 1-10 | *Alopecurus aequalis* | Poales | Poaceae | *Alopecurus* |
| 3 | 1-11 | *Coix lacryma-jobi* | Poales | Poaceae | *Coix* |
| 4 | 1-12 | *Phoebe sheareri* | Laurales | Lauraceae | *Phoebe* |
| 5 | 1-13 | *Magnolia grandiflora* | Magnoliales | Magnoliaceae | *Magnolia* |
| 6 | 1-14 | *Platycladus orientalis* | - | Cupressaceae | *Platycladus* |
| 7 | 1-2 | *Artemisia caruifolia* | Asterales | Asteraceae | *Artemisia* |
| 8 | 1-3 | *Arthraxon hispidus* | Poales | Poaceae | *Arthraxon* |
| 9 | 1-4 | *Helianthus tuberosus* | Asterales | Asteraceae | *Helianthus* |
| 10 | 1-5 | *Bidens pilosa* | Asterales | Asteraceae | *Bidens* |
| 11 | 1-6 | *Rhamnus utilis* | Rosales | Rhamnaceae | *Rhamnus* |
| 12 | 1-7 | *Zelkova serrata* | Rosales | Ulmaceae | *Zelkova* |
| 13 | 1-8 | *Pyrus xerophila* | Rosales | Rosaceae | *Pyrus* |
| 14 | 1-9 | *Causonis japonica* | Vitales | Vitaceae | *Causonis* |
| 15 | 2-1 | *Woodwardia japonica* | Polypodiales | Blechnaceae | *Woodwardia* |
| 16 | 2-2 | *Artemisia hedinii* | Asterales | Asteraceae | *Artemisia* |
| 17 | 2-3 | *Dicranopteris pedata* | Polypodiales | Gleicheniaceae | *Dicranopteris* |
| 18 | 2-4 | *Smilax discotis* | Liliales | Smilacaceae | *Smilax* |
| 19 | 2-6 | *Smilax davidiana* | Liliales | Smilacaceae | *Smilax* |
| 20 | 2-7 | *Cyrtomium caryotideum* | Polypodiales | Dryopteridaceae | *Cyrtomium* |
| 21 | 3-1 | *Dalbergia hupeana* | Fabales | Fabaceae | *Dalbergia* |
| 22 | 3-2 | *Platanus orientalis* | Proteales | Platanaceae | *Platanus* |
| 23 | 3-3 | *Cunninghamia lanceolata* | - | Cupressaceae | *Cunninghamia* |
| 24 | 3-35 | *Mallotus japonicus* | Malpighiales | Euphorbiaceae | *Mallotus* |
| 25 | 3-4 | *Prunus salicina* | Rosales | Rosaceae | *Prunus* |
| 26 | 3-42 | *Stephania tetrandra* | Ranunculales | Menispermaceae | *Stephania* |
| 27 | 3-43 | *Hypericum monogynum** | Malpighiales | Hypericaceae | *Hypericum* |
| 28 | 3-45 | *Boehmeria nivea* | Rosales | Urticaceae | *Boehmeria* |
| 29 | 3-47 | *Abelia chinensis* | Dipsacales | Caprifoliaceae | *Abelia* |
| 30 | 3-48 | *Artemisia mongolica* | Asterales | Asteraceae | *Artemisia* |
| 31 | 3-5 | *Eucommia ulmoides* | Garryales | Eucommiaceae | *Eucommia* |
| 32 | 3-64 | *Alangium chinense* | Cornales | Cornaceae | *Alangium* |
| 33 | 3-65 | *Mallotus apelta* | Malpighiales | Euphorbiaceae | *Mallotus* |
| 34 | 3-66 | *Phyllostachys edulis* | Poales | Poaceae | *Phyllostachys* |
| 35 | 3-67 | *Lespedeza bicolor* | Fabales | Fabaceae | *Lespedeza* |
| 36 | 3-9 | *Pueraria montana* | Fabales | Fabaceae | *Pueraria* |
| 37 | 4-1 | *Artemisia argyi* | Asterales | Asteraceae | *Artemisia* |
| 38 | 4-10 | *Camellia oleifera* | Ericales | Theaceae | *Camellia* |
| 39 | 4-11 | *Asparagus cochinchinensis* | Asparagales | Asparagaceae | *Asparagus* |
| 40 | 4-12 | *Rhus chinensis* | Sapindales | Anacardiaceae | *Rhus* |
| 41 | 4-13 | *Solanum melongena* | Solanales | Solanaceae | *Solanum* |
| 42 | 4-14 | *Phyllanthus urinaria* | Malpighiales | Phyllanthaceae | *Phyllanthus* |
| 43 | 4-15 | *Ligustrum lucidum* | Lamiales | Oleaceae | *Ligustrum* |
| 44 | 4-16 | *Matthiola incana* | Brassicales | Brassicaceae | *Matthiola* |
| 45 | 4-19 | *Perilla frutescens* | Lamiales | Lamiaceae | *Perilla* |
| 46 | 4-2 | *Osmanthus fragrans* | Lamiales | Oleaceae | *Osmanthus* |
| 47 | 4-20 | *Ampelopsis glandulosa** | Vitales | Vitaceae | *Ampelopsis* |
| 48 | 4-21 | *Clinopodium chinense* | Lamiales | Lamiaceae | *Clinopodium* |
| 49 | 4-23 | *Solanum aculeatissimum* | Solanales | Solanaceae | *Solanum* |
| 50 | 4-24 | *Cephalostachyum pergracile* | Poales | Poaceae | *Cephalostachyum* |
| 51 | 4-25 | *Quercus fabri* | Fagales | Fagaceae | *Quercus* |
| 52 | 4-26 | *Paederia foetida* | Gentianales | Rubiaceae | *Paederia* |
| 53 | 4-27 | *Imperata cylindrica* | Poales | Poaceae | *Imperata* |
| 54 | 4-28 | *Momordica charantia* | Cucurbitales | Cucurbitaceae | *Momordica* |
| 55 | 4-3 | *Triadica sebifera* | Malpighiales | Euphorbiaceae | *Triadica* |
| 56 | 4-30 | *Amaranthus hybridus* | Caryophyllales | Amaranthaceae | *Amaranthus* |
| 57 | 4-31 | *Sesamum indicum* | Lamiales | Pedaliaceae | *Sesamum* |
| 58 | 4-32 | *Phyllostachys sulphurea* | Poales | Poaceae | *Phyllostachys* |
| 59 | 4-33 | *Uncaria rhynchophylla* | Gentianales | Rubiaceae | *Uncaria* |
| 60 | 4-34 | *Aster tataricus* | Asterales | Asteraceae | *Aster* |
| 61 | 4-35 | *Oplismenus compositus* | Poales | Poaceae | *Oplismenus* |
| 62 | 4-37 | *Prunus serrulata* | Rosales | Rosaceae | *Prunus* |
| 63 | 4-38 | *Rosa laevigata* | Rosales | Rosaceae | *Rosa* |
| 64 | 4-39 | *Pennisetum alopecuroides* | Poales | Poaceae | *Pennisetum* |
| 65 | 4-4 | *Castanea seguinii* | Fagales | Fagaceae | *Castanea* |
| 66 | 4-41 | *Pinus massoniana* | - | Pinaceae | *Pinus* |
| 67 | 4-43 | *Ligustrum compactum* | Lamiales | Oleaceae | *Ligustrum* |
| 68 | 4-44 | *Potentilla freyniana* | Rosales | Rosaceae | *Potentilla* |
| 69 | 4-47 | *Prunus buergeriana* | Rosales | Rosaceae | *Prunus* |
| 70 | 4-5 | *Lysimachia congestiflora* | Ericales | Primulaceae | *Lysimachia* |
| 71 | 4-6 | *Camphora officinarum* | Laurales | Lauraceae | *Camphora* |
| 72 | 4-7 | *Setaria palmifolia* | Poales | Poaceae | *Setaria* |
| 73 | 4-8 | *Mirabilis jalapa* | Caryophyllales | Nyctaginaceae | *Mirabilis* |
| 74 | 4-9 | *Toona sinensis* | Sapindales | Meliaceae | *Toona* |
| 75 | 5-1 | *Sabia schumanniana* | Proteales | Sabiaceae | *Sabia* |
| 76 | 5-11 | *Castanopsis sclerophylla* | Fagales | Fagaceae | *Castanopsis* |
| 77 | 5-12 | *Xanthium strumarium* | Asterales | Asteraceae | *Xanthium* |
| 78 | 5-13 | *Salix babylonica* | Malpighiales | Salicaceae | *Salix* |
| 79 | 5-14 | *Dioscorea alata* | Dioscoreales | Dioscoreaceae | *Dioscorea* |
| 80 | 5-15 | *Cupressus funebris* | - | Cupressaceae | *Cupressus* |
| 81 | 5-16 | *Pterocarya stenoptera* | Fagales | Juglandaceae | *Pterocarya* |
| 82 | 5-17 | *Vernicia fordii* | Malpighiales | Euphorbiaceae | *Vernicia* |
| 83 | 5-18 | *Castanea mollissima* | Fagales | Fagaceae | *Castanea* |
| 84 | 5-19 | *Eriobotrya japonica* | Rosales | Rosaceae | *Eriobotrya* |
| 85 | 5-2 | *Ficus pumila** | Rosales | Moraceae | *Ficus* |
| 86 | 5-20 | *Juniperus formosana* | - | Cupressaceae | *Juniperus* |
| 87 | 5-21 | *Osmanthus fragrans* | Lamiales | Oleaceae | *Osmanthus* |
| 88 | 5-22 | *Phragmites australis* | Poales | Poaceae | *Phragmites* |
| 89 | 5-23 | *Ilex chinensis* | Aquifoliales | Aquifoliaceae | *Ilex* |
| 90 | 5-3 | *Colocasia esculenta* | Alismatales | Araceae | *Colocasia* |
| 91 | 5-4 | *Ziziphus jujuba* | Rosales | Rhamnaceae | *Ziziphus* |
| 92 | 5-5 | *Persicaria capitata* | Caryophyllales | Polygonaceae | *Persicaria* |
| 93 | 5-6 | *Galium spurium* | Gentianales | Rubiaceae | *Galium* |
| 94 | 5-7 | *Cycas revoluta* | Cycadales | Cycadaceae | *Cycas* |
| 95 | 5-8 | *Camellia japonica* | Ericales | Theaceae | *Camellia* |
| 96 | 5-9 | *Yulania denudata* | Magnoliales | Magnoliaceae | *Yulania* |
| 97 | 6-0 | *Lactuca sativa* | Asterales | Asteraceae | *Lactuca* |
| 98 | 6-1 | *Castanopsis tibetana* | Fagales | Fagaceae | *Castanopsis* |
| 99 | 6-10 | *Diospyros kaki* | Ericales | Ebenaceae | *Diospyros* |
| 100 | 6-11 | *Serissa japonica* | Gentianales | Rubiaceae | *Serissa* |
| 101 | 6-12 | *Acer palmatum* | Sapindales | Sapindaceae | *Acer* |
| 102 | 6-13 | *Lysimachia fortunei* | Ericales | Primulaceae | *Lysimachia* |
| 103 | 6-14 | *Carex cruciata* | Poales | Cyperaceae | *Carex* |
| 104 | 6-15 | *Symplocos tanakana* | Ericales | Symplocaceae | *Symplocos* |
| 105 | 6-16 | *Mallotus repandus* | Malpighiales | Euphorbiaceae | *Mallotus* |
| 106 | 6-17 | *Smilax glabra* | Liliales | Smilacaceae | *Smilax* |
| 107 | 6-18 | *Photinia × fraseri* | Rosales | Rosaceae | *Photinia* |
| 108 | 6-19 | *Dumasia truncata* | Fabales | Fabaceae | *Dumasia* |
| 109 | 6-2 | *Ipomoea triloba* | Solanales | Convolvulaceae | *Ipomoea* |
| 110 | 6-20 | *Rhododendron simsii* | Ericales | Ericaceae | *Rhododendron* |
| 111 | 6-21 | *Lindera aggregata* | Laurales | Lauraceae | *Lindera* |
| 112 | 6-22 | *Citrus maxima* | Sapindales | Rutaceae | *Citrus* |
| 113 | 6-23 | *Citrus japonica* | Sapindales | Rutaceae | *Citrus* |
| 114 | 6-24 | *Lagerstroemia indica* | Myrtales | Lythraceae | *Lagerstroemia* |
| 115 | 6-25 | *Morus alba* | Rosales | Moraceae | *Morus* |
| 116 | 6-26 | *Cynodon dactylon* | Poales | Poaceae | *Cynodon* |
| 117 | 6-27 | *Rhododendron simsii*(Artificial planting) | Ericales | Ericaceae | *Rhododendron* |
| 118 | 6-28 | *Callicarpa bodinieri* | Lamiales | Lamiaceae | *Callicarpa* |
| 119 | 6-29 | *Ophiopogon bodinieri* | Asparagales | Asparagaceae | *Ophiopogon* |
| 120 | 6-3 | *Prunus spinosa* | Rosales | Rosaceae | *Prunus* |
| 121 | 6-30 | *Lactuca serriola* | Asterales | Asteraceae | *Lactuca* |
| 122 | 6-31 | *Typha orientalis* | Poales | Typhaceae | *Typha* |
| 123 | 6-32 | *Lagerstroemia indica* | Myrtales | Lythraceae | *Lagerstroemia* |
| 124 | 6-33 | *Citrus sinensis* | Sapindales | Rutaceae | *Citrus* |
| 125 | 6-34 | *Ampelopsis humulifolia* | Vitales | Vitaceae | *Ampelopsis* |
| 126 | 6-35 | *Lagerstroemia micrantha* | Myrtales | Lythraceae | *Lagerstroemia* |
| 127 | 6-36 | *Persicaria perfoliata* | Caryophyllales | Polygonaceae | *Persicaria* |
| 128 | 6-37 | *Vitis bryoniifolia* | Vitales | Vitaceae | *Vitis* |
| 129 | 6-38 | *Citrus trifoliata* | Sapindales | Rutaceae | *Citrus* |
| 130 | 6-39 | *Prunus cerasifera* | Rosales | Rosaceae | *Prunus* |
| 131 | 6-4 | *Eurya nitida* | Ericales | Pentaphylacaceae | *Eurya* |
| 132 | 6-40 | *Michelia maudiae* | Magnoliales | Magnoliaceae | *Michelia* |
| 133 | 6-41 | *Musa basjoo* | Zingiberales | Musaceae | *Musa* |
| 134 | 6-42 | *Patrinia villosa* | Dipsacales | Caprifoliaceae | *Patrinia* |
| 135 | 6-43 | *Ageratum conyzoides* | Asterales | Asteraceae | *Ageratum* |
| 136 | 6-44 | *Clerodendrum cyrtophyllum* | Lamiales | Lamiaceae | *Clerodendrum* |
| 137 | 6-5 | *Citrus reticulata* | Sapindales | Rutaceae | *Citrus* |
| 138 | 6-6 | *Lonicera japonica** | Dipsacales | Caprifoliaceae | *Lonicera* |
| 139 | 6-7 | *Ampelopsis glandulosa* | Vitales | Vitaceae | *Ampelopsis* |
| 140 | 6-8 | *Symplocos sumuntia* | Ericales | Symplocaceae | *Symplocos* |
| 141 | 6-9 | *Viburnum dilatatum* | Dipsacales | Adoxaceae | *Viburnum* |
| 142 | 7-1 | *Pistacia chinensis* | Sapindales | Anacardiaceae | *Pistacia* |
| 143 | 7-10 | *Canna indica* | Zingiberales | Cannaceae | *Canna* |
| 144 | 7-11 | *Cyperus rotundus* | Poales | Cyperaceae | *Cyperus* |
| 145 | 7-12 | *Veronica polita* | Lamiales | Plantaginaceae | *Veronica* |
| 146 | 7-15 | *Rubia cordifolia* | Gentianales | Rubiaceae | *Rubia* |
| 147 | 7-16 | *Salvia plebeia* | Lamiales | Lamiaceae | *Salvia* |
| 148 | 7-17 | *Lonicera japonica** | Dipsacales | Caprifoliaceae | *Lonicera* |
| 149 | 7-18 | *Luffa aegyptiaca* | Cucurbitales | Cucurbitaceae | *Luffa* |
| 150 | 7-2 | *Bambusa multiplex cv. Fernleaf* | Poales | Poaceae | *Bambusa* |
| 151 | 7-20 | *Trichosanthes kirilowii** | Cucurbitales | Cucurbitaceae | *Trichosanthes* |
| 152 | 7-21 | *Trichosanthes kirilowii** | Cucurbitales | Cucurbitaceae | *Trichosanthes* |
| 153 | 7-22 | *Biancaea decapetala* | Fabales | Fabaceae | *Biancaea* |
| 154 | 7-25 | *Oenanthe javanica* | Apiales | Apiaceae | *Oenanthe* |
| 155 | 7-26 | *Lactuca raddeana* | Asterales | Asteraceae | *Lactuca* |
| 156 | 7-27 | *Fargesia spathacea* | Poales | Poaceae | *Fargesia* |
| 157 | 7-28 | *Gossypium hirsutum* | Malvales | Malvaceae | *Gossypium* |
| 158 | 7-29 | *Sambucus javanica* | Dipsacales | Adoxaceae | *Sambucus* |
| 159 | 7-3 | *Juncus effusus* | Poales | Juncaceae | *Juncus* |
| 160 | 7-30 | *Setaria plicata* | Poales | Poaceae | *Setaria* |
| 161 | 7-32 | *Buddleja lindleyana* | Lamiales | Scrophulariaceae | *Buddleja* |
| 162 | 7-33 | *Reynoutria japonica* | Caryophyllales | Polygonaceae | *Reynoutria* |
| 163 | 7-34 | *Trema cannabina* | Rosales | Cannabaceae | *Trema* |
| 164 | 7-35 | *Gonostegia hirta* | Rosales | Urticaceae | *Gonostegia* |
| 165 | 7-36 | *Physalis philadelphica* | Solanales | Solanaceae | *Physalis* |
| 166 | 7-37 | *Acer buergerianum* | Sapindales | Sapindaceae | *Acer* |
| 167 | 7-38 | *Anthriscus sylvestris* | Apiales | Apiaceae | *Anthriscus* |
| 168 | 7-4 | *Tagetes erecta* | Asterales | Asteraceae | *Tagetes* |
| 169 | 7-40 | *Houttuynia cordata* | Piperales | Saururaceae | *Houttuynia* |
| 170 | 7-41 | *Sabia japonica* | Proteales | Sabiaceae | *Sabia* |
| 171 | 7-43 | *Paulownia tomentosa* | Lamiales | Paulowniaceae | *Paulownia* |
| 172 | 7-44 | *Catharanthus roseus* | Gentianales | Apocynaceae | *Catharanthus* |
| 173 | 7-45 | *Parthenocissus quinquefolia* | Vitales | Vitaceae | *Parthenocissus* |
| 174 | 7-46 | *Salix wilsonii* | Malpighiales | Salicaceae | *Salix* |
| 175 | 7-47 | *Glandularia × hybrida* | Lamiales | Verbenaceae | *Glandularia* |
| 176 | 7-48 | *Ligustrum sinense* | Lamiales | Oleaceae | *Ligustrum* |
| 177 | 7-50 | *Euphorbia humifusa* | Malpighiales | Euphorbiaceae | *Euphorbia* |
| 178 | 7-52 | *Celosia cristata* | Caryophyllales | Amaranthaceae | *Celosia* |
| 179 | 7-53 | *Schisandra chinensis* | Austrobaileyales | Schisandraceae | *Schisandra* |
| 180 | 7-54 | *Aster oliganthus* | Asterales | Asteraceae | *Aster* |
| 181 | 7-58 | *Daphne genkwa* | Malvales | Thymelaeaceae | *Daphne* |
| 182 | 7-59 | *Salix pseudotangii* | Malpighiales | Salicaceae | *Salix* |
| 183 | 7-6 | *Nerium oleander* | Gentianales | Apocynaceae | *Nerium* |
| 184 | 7-60 | *Blumea sinuata* | Asterales | Asteraceae | *Blumea* |
| 185 | 7-63 | *Ranunculus cantoniensis* | Ranunculales | Ranunculaceae | *Ranunculus* |
| 186 | 7-64 | *Nekemias cantoniensis* | Vitales | Vitaceae | *Nekemias* |
| 187 | 7-66 | *Celtis biondii* | Rosales | Ulmaceae | *Celtis* |
| 188 | 7-67 | *Photinia prunifolia* | Rosales | Rosaceae | *Photinia* |
| 189 | 7-68 | *Lamium barbatum* | Lamiales | Lamiaceae | *Lamium* |
| 190 | 7-7 | *Celosia argentea* | Caryophyllales | Amaranthaceae | *Celosia* |
| 191 | 7-8 | *Flueggea suffruticosa* | Malpighiales | Phyllanthaceae | *Flueggea* |
| 192 | 8-1 | *Agrimonia pilosa* | Rosales | Rosaceae | *Agrimonia* |
| 193 | 8-10 | *Justicia procumbens* | Lamiales | Acanthaceae | *Justicia* |
| 194 | 8-11 | *Euonymus alatus* | Celastrales | Celastraceae | *Euonymus* |
| 195 | 8-12 | *Ardisia crenata* | Ericales | Primulaceae | *Ardisia* |
| 196 | 8-13 | *Carpesium abrotanoides* | Asterales | Asteraceae | *Carpesium* |
| 197 | 8-14 | *Pilea notata* | Rosales | Urticaceae | *Pilea* |
| 198 | 8-15 | *Ficus pumila* | Rosales | Moraceae | *Ficus* |
| 199 | 8-17 | *Akebia trifoliata* | Ranunculales | Lardizabalaceae | *Akebia* |
| 200 | 8-18 | *Kadsura longipedunculata* | Austrobaileyales | Schisandraceae | *Kadsura* |
| 201 | 8-23 | *Euscaphis japonica* | Crossosomatales | Staphyleaceae | *Euscaphis* |
| 202 | 8-24 | *Rosa multiflora* | Rosales | Rosaceae | *Rosa* |
| 203 | 8-26 | *Erigeron canadensis* | Asterales | Asteraceae | *Erigeron* |
| 204 | 8-27 | *Cyperus michelianus* | Poales | Cyperaceae | *Cyperus* |
| 205 | 8-28 | *Melia azedarach* | Sapindales | Meliaceae | *Melia* |
| 206 | 8-29 | *Akebia trifoliata* | Ranunculales | Lardizabalaceae | *Akebia* |
| 207 | 8-3 | *Pleuropterus multiflorus** | Caryophyllales | Polygonaceae | *Pleuropterus* |
| 208 | 8-31 | *Cocculus orbiculatus* | Ranunculales | Menispermaceae | *Cocculus* |
| 209 | 8-32 | *Dioscorea polystachya* | Dioscoreales | Dioscoreaceae | *Dioscorea* |
| 210 | 8-33 | *Tubocapsicum anomalum* | Solanales | Solanaceae | *Tubocapsicum* |
| 211 | 8-34 | *Salvia chinensis* | Lamiales | Lamiaceae | *Salvia* |
| 212 | 8-36 | *Litsea cubeba* | Laurales | Lauraceae | *Litsea* |
| 213 | 8-37 | *Tripterospermum chinense* | Gentianales | Gentianaceae | *Tripterospermum* |
| 214 | 8-38 | *Smilax glaucochina* | Liliales | Smilacaceae | *Smilax* |
| 215 | 8-39 | *Stephania cephalantha* | Ranunculales | Menispermaceae | *Stephania* |
| 216 | 8-4 | *Lysimachia patungensis* | Ericales | Primulaceae | *Lysimachia* |
| 217 | 8-40 | *Elsholtzia argyi* | Lamiales | Lamiaceae | *Elsholtzia* |
| 218 | 8-41 | *Hypericum monogynum* | Malpighiales | Hypericaceae | *Hypericum* |
| 219 | 8-42 | *Distylium buxifolium* | Saxifragales | Hamamelidaceae | *Distylium* |
| 220 | 8-43 | *Ainsliaea glabra* | Asterales | Asteraceae | *Ainsliaea* |
| 221 | 8-45 | *Podocarpus macrophyllus* | Podocarpales | Podocarpaceae | *Podocarpus* |
| 222 | 8-46 | *Ardisia escallonioides* | Ericales | Primulaceae | *Ardisia* |
| 223 | 8-47 | *Artemisia annua* | Asterales | Asteraceae | *Artemisia* |
| 224 | 8-48 | *Syzygium buxifolium* | Myrtales | Myrtaceae | *Syzygium* |
| 225 | 8-49 | *Mussaenda shikokiana* | Gentianales | Rubiaceae | *Mussaenda* |
| 226 | 8-5 | *Zanthoxylum ailanthoides* | Sapindales | Rutaceae | *Zanthoxylum* |
| 227 | 8-50 | *Corydalis pallida* | Ranunculales | Papaveraceae | *Corydalis* |
| 228 | 8-51 | *Zanthoxylum armatum* | Sapindales | Rutaceae | *Zanthoxylum* |
| 229 | 8-52 | *Salvia miltiorrhiza* | Lamiales | Lamiaceae | *Salvia* |
| 230 | 8-54 | *Alternanthera philoxeroides* | Caryophyllales | Amaranthaceae | *Alternanthera* |
| 231 | 8-55 | *Sargentodoxa cuneata* | Ranunculales | Lardizabalaceae | *Sargentodoxa* |
| 232 | 8-56 | *Mucuna sempervirens* | Fabales | Fabaceae | *Mucuna* |
| 233 | 8-58 | *Rhynchosia volubilis* | Fabales | Fabaceae | *Rhynchosia* |
| 234 | 8-59 | *Eupatorium japonicum* | Asterales | Asteraceae | *Eupatorium* |
| 235 | 8-6 | *Ardisia japonica* | Ericales | Primulaceae | *Ardisia* |
| 236 | 8-60 | *Hedera nepalensis* | Apiales | Araliaceae | *Hedera* |
| 237 | 8-61 | *Symplocos stellaris* | Ericales | Symplocaceae | *Symplocos* |
| 238 | 8-62 | *Potentilla kleiniana* | Rosales | Rosaceae | *Potentilla* |
| 239 | 8-63 | *Oreocnide frutescens* | Rosales | Urticaceae | *Oreocnide* |
| 240 | 8-64 | *Ampelopsis glandulosa* | Vitales | Vitaceae | *Ampelopsis* |
| 241 | 8-66 | *Erigeron sumatrensis* | Asterales | Asteraceae | *Erigeron* |
| 242 | 8-67 | *Eclipta prostrata* | Asterales | Asteraceae | *Eclipta* |
| 243 | 8-68 | *Ilex verticillata* | Aquifoliales | Aquifoliaceae | *Ilex* |
| 244 | 8-69 | *Solanum americanum* | Solanales | Solanaceae | *Solanum* |
| 245 | 8-7 | *Rubus hirsutus* | Rosales | Rosaceae | *Rubus* |
| 246 | 8-70 | *Trigonotis peduncularis* | Boraginales | Boraginaceae | *Trigonotis* |
| 247 | 8-72 | *Hedyotis chrysotricha** | Gentianales | Rubiaceae | *Hedyotis* |
| 248 | 8-73 | *Hedyotis chrysotricha** | Gentianales | Rubiaceae | *Hedyotis* |
| 249 | 8-8 | *Persicaria criopolitana* | Caryophyllales | Polygonaceae | *Persicaria* |
| 250 | 8-9 | *Ficus sarmentosa* | Rosales | Moraceae | *Ficus* |
| 251 | 9-1 | *Ligustrum vulgare* | Lamiales | Oleaceae | *Ligustrum* |
| 252 | 9-11 | *Urena lobata* | Malvales | Malvaceae | *Urena* |
| 253 | 9-12 | *Ipomoea batatas* | Solanales | Convolvulaceae | *Ipomoea* |
| 254 | 9-13 | *Pyracantha fortuneana* | Rosales | Rosaceae | *Pyracantha* |
| 255 | 9-15 | *Quercus phellos* | Fagales | Fagaceae | *Quercus* |
| 256 | 9-16 | *Mosla scabra* | Lamiales | Lamiaceae | *Mosla* |
| 257 | 9-17 | *Deutzia crenata* | Cornales | Hydrangeaceae | *Deutzia* |
| 258 | 9-19 | *Glycine max* | Fabales | Fabaceae | *Glycine* |
| 259 | 9-2 | *Lactuca indica* | Asterales | Asteraceae | *Lactuca* |
| 260 | 9-20 | *Sorghum bicolor* | Poales | Poaceae | *Sorghum* |
| 261 | 9-3 | *Pleuropterus multiflorus** | Caryophyllales | Polygonaceae | *Pleuropterus* |
| 262 | 9-4 | *Ampelopsis glandulosa* | Vitales | Vitaceae | *Ampelopsis* |
| 263 | 9-6 | *Coriandrum sativum* | Apiales | Apiaceae | *Coriandrum* |
| 264 | 9-7 | *Salix rosthornii* | Malpighiales | Salicaceae | *Salix* |
| 265 | 9-8 | *Ilex aculeolata* | Aquifoliales | Aquifoliaceae | *Ilex* |
| 266 | 9-9 | *Corydalis incisa* | Ranunculales | Papaveraceae | *Corydalis* |
| 267 | 10-11 | *Ipomoea hederifolia* | Solanales | Convolvulaceae | *Ipomoea* |
| 268 | 10-12 | *Rorippa cantoniensis** | Brassicales | Brassicaceae | *Rorippa* |
| 269 | 10-13 | *Allium fistulosum* | Asparagales | Amaryllidaceae | *Allium* |
| 270 | 10-14 | *Rorippa cantoniensis** | Brassicales | Brassicaceae | *Rorippa* |
| 271 | 10-15 | *Koelreuteria paniculata* | Sapindales | Sapindaceae | *Koelreuteria* |
| 272 | 10-16 | *Aloe vera* | Asparagales | Asphodelaceae | *Aloe* |
| 273 | 10-17 | *Prunus persica* | Rosales | Rosaceae | *Prunus* |
| 274 | 10-18 | *Mentha spicata* | Lamiales | Lamiaceae | *Mentha* |
| 275 | 10-19 | *Trachelospermum jasminoides* | Gentianales | Apocynaceae | *Trachelospermum* |
| 276 | 10-20 | *Alcea rosea* | Malvales | Malvaceae | *Alcea* |
| 277 | 10-21 | *Nandina domestica* | Ranunculales | Berberidaceae | *Nandina* |
| 278 | 10-22 | *Zea mays* | Poales | Poaceae | *Zea* |
| 279 | 10-23 | *Michelia figo* | Magnoliales | Magnoliaceae | *Michelia* |
| 280 | 10-24 | *Euonymus maackii* | Celastrales | Celastraceae | *Euonymus* |
| 281 | 10-25 | *Ulmus pumila* | Rosales | Ulmaceae | *Ulmus* |
| 282 | 10-26 | *Clematis terniflora* | Ranunculales | Ranunculaceae | *Clematis* |
| 283 | 10-27 | *Alnus cremastogyne* | Fagales | Betulaceae | *Alnus* |
| 284 | 10-29 | *Gomphrena globosa* | Caryophyllales | Amaranthaceae | *Gomphrena* |
| 285 | 10-30 | *Yulania liliiflora* | Magnoliales | Magnoliaceae | *Yulania* |
| 286 | 10-6 | *Rosa chinensis* | Rosales | Rosaceae | *Rosa* |
| 287 | 12-1 | *Loropetalum chinense* | Saxifragales | Hamamelidaceae | *Loropetalum* |
| 288 | 12-3 | *Ixeris japonica* | Asterales | Asteraceae | *Ixeris* |
| 289 | 12-4 | *Lysimachia christinae* | Ericales | Primulaceae | *Lysimachia* |
| 290 | 12-6 | *Clematis puberula* | Ranunculales | Ranunculaceae | *Clematis* |

Table Note: “*” indicates the collection of duplicate plant samples(8); - indicates an unknown.
