# Supplementary material for: Assembling and dietary application of a local trnL metabarcoding database for Cervusnipponkopschi in Taohongling Nature Reserve
Source: Biodivers Data J. 2024 Nov 21;12:e139269. doi: 10.3897/BDJ.12.e139269 (PMC11605298; doi:10.3897/BDJ.12.e139269)
Supplement: Supplementary material 2 — Local reference database [file bdj-12-e139269-s002.docx]

**Supplementary material table S2.** The local reference database based on the trnL gene (containing 162 plants’ DNA sequence information)

| **Number** | **Name** | **Potential forage plants** | **Sequence information** |
| --- | --- | --- | --- |
| 1 | 10-12 | *Rorippa cantoniensis** | TCCATTGAGTCTCTGCACCTATCCCTTTTTTTTTCTCGCTTTCTAAACCGGGGTTTGTTCGCGTAAACAAGGATTTGGCTCAGGATTGCCCATTGTTAATTCCAGGGTTTCTCTGAATTTGAAAGTTATCACTTAGTAGGTTTCCATACCAAGGCTCAATCCAATTAAGTCCGTAGCGTCTACCGATTTCGA |
| 2 | 10-13 | *Allium fistulosum* | TCCATTGAGTCTCTGCACCTATCCTTTTTTATTCTCTTTTTTAAACCCTTGTTTTTCAAAAAATAAAGATTTGGCTCAGGATTGCCCATTTTTAGTTCCAGGGTTTCTCTGAATTTGGAAGTTTTCACTTAGCAGGTTTCCATACTAAGGCTCAATCCAATCAAGTCCGTAGCGTCTACCGATTTCGA |
| 3 | 10-14 | *Rorippa cantoniensis** | TCCATTGAGTCTCTGCACCTATCCCTTTTTTTTTCTCGCTTTCTAAACCGGGGTTTGTTCGCGTAAACAAGGATTTGGCTCAGGATTGCCCATTGTTAATTCCAGGGTTTCTCTGAATTTGAAAGTTATCACTTAGTAGGTTTCCATACCAAGGCTCAATCCAATTAAGTCCGTAGCGTCTACCGATTTCGA |
| 4 | 10-16 | *Aloe vera* | TCCATTGAGTCTCTGCACCTATCCCTTTTTTTTTCTCGCTTTCTAAACCGGGGTTTGTTCGCGTAAACAAGGATTTGGCTCAGGATTGCCCATTGTTAATTCCAGGGTTTCTCTGAATTTGAAAGTTATCACTTAGTAGGTTTCCATACCAAGGCTCAATCCAATTAAGTCCGTAGCGTCTACCGATTTCGA |
| 5 | 10-17 | *Prunus persica* | TCCATTGAGTCTCTGCACCTATCCTTTTTTATTCTCGGTTTATGAAACCCTTGTTTGTTTTAATAAAACAGGATTTGGCTCAGGATCGCCCATTTTTAATTCCAGGGTTTCTCTGAATTTGAAAGTTCTCACTTGGTAGGTTTCCATACCAAGGCTCAATCCAATTAAGTCCGTAGCGTCTACCGATTTCGA |
| 6 | 10-19 | *Trachelospermum jasminoides* | TCCATTGAGTCTCTGCACCTATCCTTGTTTTCGTTTTCTGAACCTTTGTTTGTGGAAAATAGGATTTGGCTCAGGATTGCCCTTTTTCTTAATTCCGGGGTTTCTCTGAATTTGAAAGTCATCACTTAGTAGGTTTCCTTACCAAGGCTCAATCCAATTAAGTCCGTAGCGTCTACCGATTTCGAAACG |
| 7 | 10-21 | *Nandina domestica* | TCCATTGAGTCTCTGCACCTATCCCTTTTTTCTGAAAACAGGATTTGGCTCAGGATTGCCCATTTTTAATTCCAGGGTTTCTCTGAATTTGAAAGTTATCACTTAGTAGGTTTCCATACCAAGGCTCAATCCAATCAAGTCCGTAGCGTCTACCGATTTCGA |
| 8 | 10-22 | *Zea mays* | TCCATTGAGTCTCTGCACCTATCCTTTTCCTTTGGGTTCTAGTTTGAGAACCACTTGTTTTTCAAAAAAGGGATTTGGCTCAGGATTGCCCATTTTTCATTCCAGGGTTTCTCTGAATTTGGAAGTTACCACTTAGCAGGTTTCCATACCAAGGCTCAATACAATCAAGTCCGTAGCGTCTACCGATTTCGA |
| 9 | 10-23 | *Michelia figo* | TCGAAATCGGTAGACGCTACGGACTTGATTGGATTGAGCCTTGGTATGGAAACCTACTAAGTGGTAACTTCCAAATTCAGAGAAACCCTGGAATTAAAAATGGGTAATCCTGAGCCAAATCCTGTGTTCAAAAAACAAGGGTTCAGAAAGCGAGAATCAAAAAAGGATAGGTGCAGAGACTCAATGGA |
| 10 | 10-26 | *Clematis terniflora* | TTCCATTGAATCTTTGCCCCTATCCTTTTTTTTTTATTCTTGCTTTCTGAAGCCCTTTTGTTTTCTGAAAAAAGGATTTGGCTCAGGATTGCCCATTTTTTATTCCGGGGTTTCTCTGAATTTGAAAGTTATCACTTAGTATGTTTCCATATCAAGGCTCAATCCAACCAAGTCCGTAGCGTCTACCGATTTCGA |
| 11 | 10-29 | *Gomphrena globosa* | TCCATTGAGTCTCTGCACCTATCCTTTTATTTTTTTTATTCTTGGTTTCTGAATTCTTATTTTTTTCCTAATAAAGGAGTTGGCTCAGGATTGCCCATTTTTTTATTAATTCCAGGGTTTCTCTGAATTTGAAAGTTTTCACTTAGTAGGTCTCCATACTAAGGCTCAAGCCAATTAAGTCCGTAGCGTCTACCGATTTCGA |
| 12 | 10-30 | *Yulania liliiflora* | TCCATTGAGTCTCTGCACCTATCCTTTTTTGATTCTCGCTTTCTGAACCCTTGTTTTCTGAACACAGGATTTGGCTCAGGATTGCCCATTTTTAATTCCAGGGTTTCTCTGAATTTGGAAGTTACCACTTAGTAGGTTTCCCTACCAAGGCTCAATCCAATCAAGTCCGTAGCGTCTACCGATTTCGA |
| 13 | 1-10 | *Alopecurus aequalis* | TCCATTGAGTCTCTGCACCTATCCTTTTCCTTTGTATTCTAGTTCGATTCGAGAACCCCTTTGTTTTCTCAAAACACGGATTTGGCTCAGGATTGCCCTTTTTTAATTCCAGGGTTTCTCTGAATTTGGAAGTTACCACTTAGCAGGTTTCCATACCAAGGCTCAATACAATCAAGTCCGTAGCGTCTACCGATTTCGA |
| 14 | 1-11 | *Coix lacryma-jobi* | TCCATTGAGTCTCTGCACCTATCCTTTTTTTTATTCTAGTTTGATAAACCTTAGTTTTATCAAACTAAGGATTTGGCTCAGGATTGCCCATTTTAAATTCCAGGGTTTCTCTGAATTTGGAAGTTACCACTTAGCAGGTTTCCTTACTAAGGCTCAATCCAATCAAGTCCGTAGCGTCTACCGATTTCGA |
| 15 | 1-12 | *Phoebe sheareri* | TCCATTGAGTCTCTGCACCTATCCTTTTTTTGGTTCTCGCTTTCTGAACCCTTGTTTTCTGAAAACAGGATTTGGCTCAGGATTGCCCATTTTTAATTCCAGGGTTTCTCTGAATTTGGAAGTTATCACTTAGTAGGTTTCCATACCAAGGCTCAATCCAACCAAGTCCGTAGCGTCTACCGATTTCGAAA |
| 16 | 1-13 | *Magnolia grandiflora* | TCCATTGAGTCTCTGCACCTATCCTTTTTTGATTCCCGCTTTCTGAACCCTTGTTTTCTGAACACAGGATTTGGCTCAGGATTGCCCATTTTTAATTCCAGGGTTTCTCTGAATTTGGAAGTTACCACTTAGTAGGTTTCCATACCAAGGCTCAATCCAATCAAGTCCGTAGCGTCTACCGATTTCGAAA |
| 17 | 1-2 | *Artemisia caruifolia* | CCATTGAGTCTCTGCACCTATCTTTTTTTTCTTTTCGCTTTCTGAACCTTTGTTTGTTTTCGGAAAACGTGATTTGGCTCAGGATTGCCCATTTTTCTTAATTCCAGGGTTTCTCTGAATTTGAAAGTTATCACTTAGTAAGTTGCCATACCAAGGCTCAATCCAATTAAGTCCGTAGCGTCTACCGATTTC |
| 18 | 12-1 | *Loropetalum chinense* | TCCATTGAGTCTCTGCACCTATCCTTTTTTTATTTTGATTCTCGCTTTCTGAACCCTTGTCTTTGTTTTCGGAAAACAGGATTTGGCTCAGGATTGCCCCTTTTTAATTCCAGGGTTTCTCTGAATTTGAAAGTTATCACTTAGTAGGTTTCCATACCAAGGCTCAATCAAATTAAGTCCGTAGCGTCTACCGATTTCG |
| 19 | 12-3 | *Ixeris japonica* | TCCATTGAGTCTCTGCACCTATCCTTTTTTGATTTTCGCTTTCTGAACCTTTGTTTGTTTTCGGAAAACGTGATTTGGCTCAGGATTGCCCATTTTTATTAATTCCAGGGTTTCTCTGAATTTGAAAGTTATCACTTAGTAAGTTGCCATACCAAGGCTCAATCCAATTAAGTCCGTAGCGTCTACCGATTTCGA |
| 20 | 12-4 | *Lysimachia christinae* | TCCATTGAGTCTCTGCACCTATCCCCCTTTTTTATTTTCCTTTAAATCTTTGTTTTCGAAAAAGAGGATTTGGCTCAGGATTGCCCATTTTTATTAATTCCAGGGGTTCTCTGAATTTGAAAGTTCTCACTTAGCAGGTTTCCATACTAAGGCTCAATGCAATTAAGTCCGTAGCGTCTACCGATTTCGA |
| 21 | 1-3 | *Arthraxon hispidus* | TCCATTGAGTCTCTGCACCTATCCTTTTCCTTTGGGTTCTAGTTTAAGAACCACTTGTTTTTTCAAAAAAAGGATTTGGCTCAGGATTGCCCATTTTTCATTCCAGGGTTTCTCTGAATTTGGAAGTTACCACTTAGCAGGTTTCCATACCAAGGCTCAATACAATCAAGTCCGTAGCGTCTACCGATTTCGA |
| 22 | 2-4 | *Smilax discotis* | TCCATTGAGTCTCTGCACCTATCCCTTTTTGATTCTAGTTTTATAAATTAAACCCCTATTTTATCAAAATAAAGATTTGGCTCAGGATTGCCCATTTTGAATTCCGGGGTTTCTCTGAATTTGGAAGTTACCACTTAGCAGGTTTCCATACCAAGGCTCAAGCTAATCAAGTCCGTAGCGTCTACCGATTTCGA |
| 23 | 2-6 | *Smilax davidiana* | TCCATTGAGTCTCTGCACCTATCCCTTTTTGATTCTAGTTTTATAAATTAAACCCCTATTTTATCAAAATAAAGATTTGGCTCAGGATTGCCCATTTTGAATTCCGGGGTTTCTCTGAATTTGGAAGTTACCACTTAGCAGGTTGCCATACCAAGGCTCAAGCCAATCAAGTCCGTAGCGTCTACCGATTTCGA |
| 24 | 3-1 | *Dalbergia hupeana* | TCCATTGAGTCTCTGCACCTATCCTTTTTTTATTATCGCTTTCTTAATTTTTCTTTGCTTTCAGAAAACGGGATTTGGCTCAGGATTGCCCATTGTTAATTCCGGGGTTTCTCTGAATTTGAAAGTTATCACTTGGTACGTTGCCATACCAAGGCTCAATCCAATTAAGTCCGTAGCGTCTACCGATTTCG |
| 25 | 3-3 | *Cunninghamia lanceolata* | TCCATTGAGTCTCTGCACCTATCCCTTTCTAGGATAGGAGACTATTGTCTCCAGAAATCGGATTTGGCTCAGGATTGCCCATTCAAAATATCCCAGGGTTCCCTGGATTTGGATGCTATCACTTGGTAAGTTTCCATACCAAGGCTCAAAAAATTTAAGTCCGTAGCGTCTACCGATTTCGA |
| 26 | 3-35 | *Liquidambar formosana* | TCCATTGAGTCTCTGCACCTATCCTTTTTTTATTCTGTATTTATGAACCCTTATTTGTTTTCGTAAAACAGGATTTGGCTCAGGATTGCCCATTTTTAATTCCAGGGTTTCTCTGAATTTGAAAGTTATCACTTAGTAAGTTGCCATACCAAGGCTCAATCTAATTAAGTCCGTAGCGTCTACCGATTTCG |
| 27 | 3-4 | *Prunus salicina* | TCCATTGAGTCTCTGCACCTATCCTTTTTTATTCTCGGTTTATGAAACCCTTGTTTGTTTTAATAAAACAGGATTTGGCTCAGGATCGCCCATTTTTAATTCCAGGGTTTCTCTGAATTTGAAAGTTCTCACTTGGTAGGTTTCCATACCAAGGCTCAATCCAATTAAGTCCGTAGCGTCTACCGATTTCGAAA |
| 28 | 3-47 | *Abelia chinensis* | TCCATTGAGTCTCTGCACCTATCCTTTTTGATTTTAGCTTTCTGAACCCTTGTTTTCGTAAAACTGGATTTGGCTCAGGATTGCCCATTTTTATTAATTCCGGGGTTTCTCTGAATTTGAAAGTTCTCACTTAGTAGGTTTCCATACCAAGGCTCAATCCAATTAAGTCCGTAGCGTCTACCGATTTCG |
| 29 | 3-48 | *Artemisia mongolica* | TCCATTGAGTCTCTGCACCTATCTTTTTTTTCTTTTCGCTTTCTGAACCTTTGTTTGTTTTCGGAAAACGTGATTTGGCTCAGGATTGCCCATTTTTCTTAATTCCAGGGTTTCTCTGAATTTGAAAGTTATCACTTAGTAAGTTTCCATACCAAGGCTCAATCCAATTAAGTCCGTAGCGTCTACCGATTTCGA |
| 30 | 3-66 | *Phyllostachys edulis* | TCCATTGAGTCTCTGCACCTATCCTTTTCCTTTGGATTCTAGTTCGAGAACCACTTGTTTTCTCAAAACACGGATTTGGCTCAGGATTGCCCTTTTTTAATTCCAGGGTTTCTCTGAATTTGGAAGTTACCACTTAGCAGGTTTCCATACCAAGGCTCAATACAATCAAGTCCGTAGCGTCTACCGATTTCGA |
| 31 | 4-1 | *Artemisia stolonifera* | CCATTGAGTCTCTGCACCTATCTTTTTTTTCTTTTCGCTTTCTGAACCTTTGTTTGTTTTCGGAAAACGTGATTTGGCTCAGGATTGCCCATTTTTCTTAATTCCAGGGTTTCTCTGAATTTGAAAGTTATCACTTAGTAAGTTGCCATACCAAGGCTCAATCCAATTAAGTCCGTAGCGTCTACCGATTTCGA |
| 32 | 4-10 | *Camellia oleifera* | TCCATTGAGTCTCTGCACCTATCCTTTTTTTATTTTCGCTTTCGGAATCTTTGTTTGTTTTAGAAAAACAGGATTTGGCTCAGGATTGCCCATTTTTATTAATTCCAGGGTTTCTCTGAATTTGAAAGTTATCACTTAGTAGGTTGCCATACCAAGGCTCAATCCAATTAAGTCCGTAGCGTCTACCGATTTCGA |
| 33 | 4-12 | *Rhus chinensis* | TCCATTGAGTCTCTGCACCTATCCTCTTTCTCCCGTTCTGACCCCTTGTTTGTTTTTGTTCTCATAAAATAGGATTTGGCTCAGGATTGCCCATTTTTGATTCCAGGGTTTCTCTGAATTTGAAAGTTATCACTTAGTAGGTTTCCATACCAAGGCTCAATCCAATTAAGTCCGTAGCGTCTACCGATTTCGT |
| 34 | 4-13 | *Solanum melongena* | CCTTCCATTGAGTCTCTGCACCTATCCTTTTTTTCTGAACCTTTGTATGTTTTGAGAAAACAGGATTTGGCTCAGGATTGCCCATTTTTGTTAATTCCAGGGTTTCTCTGAATTTGAAAGTGATCACTTAGTAAGTTTCCATACCAAGGCTCAATCCAATTAAGTCCGTAGCGTCTACCGATTTCGA |
| 35 | 4-14 | *Phyllanthus urinaria* | TCCATTGAGTCTCTGCACCTATCCTTTATTTATTCTATCTTTATGATACGAACCTTTGTTTGTTTTTGGAAAACCGGATTTGGCTCAGGATTGCCCATTTTTAATTCCAGGGTTCCTCTGAATTTGAAAGTTATCACTTAGTAAGTTGCCATACCAAGGCTCAATCCAATTAAGTCCGTAGCGTCTACCGATTTCGA |
| 36 | 4-15 | *Ligustrum lucidum* | TCCATTGAGTCTCTGCACCTATCCTTTTTTCTTTCTGAACCTTTATTTTGGGAAAACAGGATTTGGCTCAGGATTGCCCATTTTTATTAATTCCGGGGTTTCTCTGAATTTGAAAGTTACCACTTAGTAGGTTGCCATACCAAGGCTCAATCCAATTAAGTCCGTAGCGTCTACCGATTTCGA |
| 37 | 4-2 | *Osmanthus fragrans* | CCCCCGTCCTCCATTGAGTCTCTGCACCTATCCTTTTTTCTTTCTGAACCTTTGTTTTGGGAAAACAGGATTTGGCTCAGGATTGCCCATTTTTATTAATTCCGGGGTTTCTCTGAATTTGAAAGTTACCACTTAGTAGGTTACCATACCAAGGCTCAATCCAATTAAGTCCGTAGCGTCTACCGATTTCGAAATG |
| 38 | 4-20 | *Ampelopsis glandulosa** | TCCATTGAGTCTCTGCACCTATCCTTTTTTTATTATCGTTTTCTGAACCCTTGGTTGTTTTCGGAAAACAGGATTTGGCTCAGGATTGCCCATTTTTTATTCCAGGGTTTCCCTGAATTTGAAAGTTATCACTTAGTAGGTTTCCATATTAAGGCTCAATCCAATTAAGTCCGTAGCGTCTACCGATTTCGA |
| 39 | 4-21 | *Clinopodium chinense* | TCCATTGAGTCTCTGCACCTATCCTTTTTTTCGTTTTTTGAACCTTTGTTTTGAGAAAACAAAAACAGGATTTGGCTCAGGATTGCCCATTTTTATTAATTCCGGGGTTTCTCTGAATTTGAAAGTTATCACTTAGTAGGTTGCCATACCAAGGCTCAATCCAATTAAGTCCGTAGCGTCTACCGATTTCGA |
| 40 | 4-26 | *Paederia foetida* | TCCATTGAGTCTCTGCACCTATCCCTTTTTTCACTTTCTGAACCTTTGTTTTCGGAAAATAGGATTTGGCTCAGGATTGCCCCTTTTTATTAATTCCAGGGTTTCTCTGAATTTGAAAGTTCTCACTCAGTGGGTTTCCATACCAAGGCCCAATCCAATTAAGTCCGTAGCGTCTACCGATTTCGA |
| 41 | 4-27 | *Imperata cylindrica* | TCCATTGAGTCTCTGCACCTATCCTTTTCCTTTGGGTTCTAGTTTGAGAACCACTTGTTTTTTCAAAAAAGTGGATTTGGCTCAGGATTGCCCATTTTTCATTCCAGGGTTTCTCTGAATTTGGAAGTTACCACTTAGCAGGTTTCCATACCAAGGCTCAATACAATCAAGTCCGTAGCGTCTACCGATTTCGA |
| 42 | 4-31 | *Sesamum indicum* | CCATCCATTGAGTCTCTGCACCTATCCTTTTTTTTCGTTTTCCGAACCTTTGTTTTGAGAAAACAGGATTTGGCTCAGGATTGCCCATTTTTATTAATTCCGGGGTTTCTCTGAATTTGAAAGTTATCACTTAGTAGGTTTCCATACCAAGGCTCAATCCAATTAAGTCCGTAGCGTCTACCGATTTCGA |
| 43 | 4-32 | *Phyllostachys sulphurea* | TCCATTGAGTCTCTGCACCTATCCTTTTCCTTTGGATTCTAGTTCGAGAACCACTTGTTTTCTCAAAACACGGATTTGGCTCAGGATTGCCCTTTTTTAATTCCAGGGTTTCTCTGAATTTGGAAGTTACCACTTAGCAGGTTTCCATACCAAGGCTCAATACAATCAAGTCCGTAGCGTCTACCGATTTCGACTCG |
| 44 | 4-37 | *Prunus serrulata* | TCCATTGAGTCTCTGCACCTATCCTTTTTTATTCTCGGTTTATGAAACCCTTGTTTGTTTTAATAAAACAGGATTTGGCTCAGGATCGCCCATTTTTAATTCCAGGGTTTCTCTGAATTTGAAAGTTCTCACTTGGTAGGTTTCCATACCAAGGCTCAATCCAATTAAGTCCGTAGCGTCTACCGATTTCGACAG |
| 45 | 4-38 | *Rosa laevigata* | TCCATTGAGTCTCTGCACCTATCCTTTATTTATTCTCGCTTTCTGAAACCTTGTTTGTTTTCATAAAACGGGATTTGGCTCAGGATTGCCCATTTTTAATTCCAGGGTTTCTCTGAATTTGAAAGTTATCACTTGGTAGGTTTCCATACCAAGGCTCAATCCAATTAAGTCCGTAGCGTCTACCGATTTCGA |
| 46 | 4-39 | *Pennisetum alopecuroides* | TCCATTGAGTCTCTGCACCTATCCTTTTCCTTTGGGTTCTAGTTTGAGAACCACTTGTTTTTTTCAAAAAGGGATTTGGCTCAGGATTGCCCATTTTTCATTCCAGGGTTTCTCTGAATTTGGAAGTTATCACTTAGCAGGTTTCCATACCAAGGCTCAATACAATCAAGTCCGTAGCGTCTACCGATTTCGA |
| 47 | 4-43 | *Ligustrum compactum* | TCCTTCCATTGAGTCTCTGCACCTATCCTTTTTTCTTTCTGAACCTTTATTTTGGGAAAACAGGATTTGGCTCAGGATTGCCCATTTTTATTAATTCCGGGGTTTCTCTGAATTTGAAAGTTACCACTTAGTAGGTTTCCATACCAAGGCTCAATCCAATTAAGTCCGTAGCGTCTACCGATTTCGAAAT |
| 48 | 4-44 | *Potentilla freyniana* | TCCATTGAGTCTCTGCACCTATCCTTTATTTATTCTCGCTTTATGAAACCCTTGTTTGTTTTCATAAAACGGGATTTGGCTCAGGATTGCCCATTTTTAATTCCAGGGTTTCTCTGAATTTGAAAGTTATCACTTGGTAGGTTTCCATACCAAGGCTCAATCCAATTAAGTCCGTAGCGTCTACCGATTTCGA |
| 49 | 4-5 | *Lysimachia congestiflora* | TCCATTGAGTCTCTGCACCTATCCCCCTTTTTTATTTTCCTTTAAATCTTTGTTTTCGAAAAAGAGGATTTGGCTCAGGATTGCCCATTTTTATTAATTCCAGGGGTTCTCTGAATTTGAAAGTTCTCACTTAGCAGGTTTCCATACTAAGGCTCAATGCAATTAAGTCCGTAGCGTCTACCGATTTCGA |
| 50 | 4-6 | *Cinnamomum camphora* | TCCATTGAGTCTCTGCACCTATCCTTTTTTTGGTTCTCGCTTTCTGAACCCTTGTTTTCTGAAAACAGGATTTGGCTCAGGATTGCCCATTTTTAATTCCAGGGTTTCTCTGAATTTGGAAGTTATCACTTAGTAGGTTTCCATACCAAGGCTCAATCCAACCAAGTCCGTAGCGTCTACCGATTTCGA |
| 51 | 4-7 | *Setaria palmifolia* | TCCATTGAGTCTCTGCACCTATCCTTTTCCTTTGGATTCTAGTTCGAGAACCACTTGTTTTCTCAAAACACGGATTTGGCTCAGGATTGCCCTTTTTTAATTCCAGGGTTTCTCTGAATTTGGAAGTTACCACTTAGCAGGTTTCCATACCAAGGCTCAATACAATCAAGTCCGTAGCGTCTACCGATTTCGA |
| 52 | 5-11 | *Castanopsis sclerophylla* | TCCATTGAGTCTCTGCACCTATCCTTTTTTTATTCTCGCTTTCTTCTGAACCCTTATTTGTTTTCGTAAAATAGGATTTGGCTCAGGATTGCCCATTTTTAATTCCAGGGTTTCTCTGAATTTGAAAGTTATCACTTGGTAAGTTTCCATACCAAGGCTCAATCCAATTAAGTCCGTAGCGTCTACCGATTTCGACACC |
| 53 | 5-14 | *Dioscorea alata* | TCCATTGAGTCTCTGCACCTATCCTTTTTGATTTTTGATTCTAGTTTGTTTTGTTTTTTTATAAACCCTTTGGTTTATAAAAAACAAAACAAATAAAGATTTGGCTCAGGATTGCCCATTTCAAATTCCAGGGTTTCTCTGAATTTGGAAGTTATCACTTAGCAGGTTTCCATACCAAGGCTCAATTCAATCAAGTCCGTAGCGTCTACCGATTTCGA |
| 54 | 5-16 | *Pterocarya stenoptera* | TCCATTGAGTCTCTGCACCTATCCTTTTGTATTCTTATTCTGTCTTTATGAACCTTTGTTTGTTTTTTTGTTTGTTTTCGAAAAATAGGATTTGGCTCAGGATTGCCCCTTTTTTTTCCGGGGTTTCTCTGAATTTGAAAGTTATCACTTAGTAAGTTTCCATACCAAGGCTCAATCCAATTAAGTCCGTAGCGTCTACCGATTTCGA |
| 55 | 5-17 | *Vernicia fordii* | TCCATTGAGTCTCTGCACCTATCCTTTTTTTTATTCTGTCCTTTTGAACCTTTGTTTGTTTTCGGAAAACAGGATTTGGCTCAGGATTGCCCATTTTTAATTCCAGGGTTTCTCTGAATTTGAAAGTTATCACTTAGTAAGTTTCCATACCAAGGCTCAATCCAATTAAGTCCGTAGCGTCTACCGATTTCGA |
| 56 | 5-19 | *Eriobotrya japonica* | TCCATTGAGTCTCTGCACCTATCCTTTTTTATTTTCGGTTTATGAAACCCTTGTTTATTTTCATAAAACAGGATTTGGCTCAGGATTGCCCATTTTTAATTCCAGGGTTTCTCTGAATTTGAAAGTTCTCACTTGGTAGGTTTCCATACCAAGGCTCAATCCAATTAAGTCCGTAGCGTCTACCGATTTCGA |
| 57 | 5-2 | *Ficus pumila** | TCCATTGAGTCTCTGCACCTATCCTTTTTTATTATCGCCTTCTGAACCCTTGTTTGTTTTCAGAAAACCGGATTTGGCTCAGGATTGCCCATTTTTAATTCCAGGGTTTCTCTGAATTTGAAAGTTATCACTTGGTAGGTTTCCATACCAACGCTCAATTCAATTCAATTAAGTCCGTAGCGTCTACCGATTTCGA |
| 58 | 5-20 | *Juniperus formosana* | TCCATTGAGTCTCTGCACCTATCCCGTTCTCGGAAAGGAAACTATTGTCTCTAGAAATCGGATTTGGCTCAGGATTGCCCATTCAAAATATCCCAGGGTTCCCTGGATTTGGATGCTATCACTTGGTAAGTTTCCATACCAAGGCTCAAAAAATTTAAGTCCGTAGCGTCTACCGATTTCGA |
| 59 | 5-21 | *Osmanthus fragrans var. thunbergii* | TCCATTGAGTCTCTGCACCTATCCTTTTTTCTTTCTGAACCTTTGTTTTGGGAAAACAGGATTTGGCTCAGGATTGCCCATTTTTATTAATTCCGGGGTTTCTCTGAATTTGAAAGTTACCACTTAGTAGGTTTCCATACCAAGGCTCAATCCAATTAAGTCCGTAGCGTCTACCGATTTCGA |
| 60 | 5-22 | *Phragmites australis* | TCCATTGAGTCTCTGCACCTATCCTTTCCCTTTGGGTTCTAGTTTGAGAACCACCTGTTTTTTCAAAAAAGGGATTTGGCTCAGGATTGCCCATTTTTAATTCCAGGGTTTCTCTGAATTTGGAAGTTACCACTTAGCAGGTTTCCATACCAAGGCTCAATACAATCAAGTCCGTAGCGTCTACCGATTTCGACTT |
| 61 | 5-5 | *Persicaria capitata* | TCCATTGAGTCTCTGCACCTATCCTTCCTTGTTTTTTTCTGTTTTTATGAAACTTTTTCTGTTTTCGGAAAACAGGATTTGGCTCAGGATTGCCCATTTTTAATTCCAGGGTTTCTCTGAATTTGAAAGTTATCACTTAGTAAGTTTCCATACCAAGGCTCAATCCAATTAAGTCCGTAGCGTCTACCGATTTCGAAATT |
| 62 | 5-6 | *Galium spurium* | TCCATTGAGTCTCTGCACCTATCCTATTTATTATTGCTTTCTGAATCCTTGTTTGTTTTCAGAAAACCGGATTTGGCTCAGGATTGCCCATTTTTTTGAATTCCAGGGTTTCTCTGAATTTGAAAGTTATCACTTGGTAGGTTTCCATACCAAGGCTCAATCCAATTAAGTCCGTAGCGTCTACCGATTTCGA |
| 63 | 5-7 | *Cycas revoluta* | TCCATTGAGTCTCTGCACCTATCCTTTCTCTTCCTAGTCAAGGAAACTATTGTCTCTGTAAACTGGATTTGGTTCAGGATTGCCCATTCTAAATGTCCTAGGGTTCCCTGGATTTGGAAGCTATCACTTGGTAGGTTTCCATACCAAGGCTCAACTCGATTAAGTCCGTAGCGTCTACCGATTTCGA |
| 64 | 5-8 | *Camellia japonica* | TCCATTGAGTCTCTGCACCTATCCTTTTTTTATTTTCGCTTTCGGAATCTTTGTTTGTTTTAGAAAAACAGGATTTGGCTCAGGATTGCCCATTTTTATTAATTCCAGGGTTTCTCTGAATTTGAAAGTTATCACTTAGTAGGTTTCCATACCAAGGCTCAATCCAATTAAGTCCGTAGCGTCTACCGATTTCGA |
| 65 | 5-9 | *Yulania denudata* | CTATTCCATTGAGTCTCTGCACCTATCCTTTTTTGATTCTCGCTTTCTGAACCCTTGTTTTCTGAACACAGGATTTGGCTCAGGATTGCCCATTTTTAATTCCAGGGTTTCTCTGAATTTGGAAGTTACCACTTAGTAGGTTTCCCTACCAAGGCTCAATCCAATCAAGTCCGTAGCGTCTACCGATTTCGAGGA |
| 66 | 6-1 | *Castanopsis tibetana* | TCCATTGAGTCTCTGCACCTATCCTTTTTTTATTCTCGCTTTCTTCTGAACCCTTATTTGTTTTCGTAAAATAGGATTTGGCTCAGGATTGCCCATTTTTAATTCCAGGGTTTCTCTGAATTTGAAAGTTATCACTTGGTAAGTTTCCATACCAAGGCTCAATCCAATTAAGTCCGTAGCGTCTACCGATTTCGACAGA |
| 67 | 6-12 | *Acer palmatum* | TGTCCATTGAGTCTCTGCACCTATCCCTTTTTTCGCTTTCTGAACCCTTGTTTGCTTTGTTTTATTCTCGTAAAACAGGATTTGGCTCAGGATTGCCCATTTTTGATTCCCGGGTTTCTCTGAATTTGAAAGTTATCACTTAGTAGGTTTCCATACCAAGGCTCAATCCAATTAAGTCCGTAGCGTCTACCGATTTCGA |
| 68 | 6-13 | *Lysimachia fortunei* | TCCATTGAGTCTCTGCACCTATCCCCTTTTTTATTTTCCTTTAAATCTTTGTTTTCGAAAAAGAGGATTTGGCTCAGGATTGCCCATTTTTATTAATTCCAGGGGTTCTCTGAATTTGAAAGTTCTCACTTAGCAGGTTTCCATACTAAGGCTCAATCCAATTAAGTCCGTAGCGTCTACCGATTTCGA |
| 69 | 6-14 | *Carex cruciata* | TCCATTGAGTCTCTGCACCTATCTTTAATATTAGACTAAGAAAAAATATTATTTCTTATCTGAAATAAGAAATATTTTATATATTTCTTTTTCTCAAAAAGAAGATTTGGCTCAGGATTGCCCATTTTTAATTCCAGGGTTTCTCTGAATTTGGAAGTTAACACTTAGCAAGTTTCCATACCAAGGCTCAATCCAATGCAAGTCCGTAGCGTCTACCGATTTCGAGA |
| 70 | 6-15 | *Symplocos tanakana* | GGGAACCTACTAAGTGATAACTTTCAAATTCAGAGAAACCCTGGAATTAATAAAAATGGGCAATCCTGAGCCAAATCCTGTTTTTAGAAAAAAAAAAAGGAAAGGGGCAGAGCCCCATGGGAAACAGGATTTGGCTCAGGATTGCCCATTTTTATTAATTCCAGGGTTTCTCTGAATTTGAAAGTTATCACTTAGTAGGTTTCCATACCAAGGCTCAATACAATTAAGTCCGTAGCGTCTACCGATTTCGAAAGA |
| 71 | 6-16 | *Mallotus repandus* | TCCATTGAGTCTCTGCACCTATCCTTTTTTTTATTCTGTATTTATGAACCCTTATTTGTTTTCGTAAAACAGGATTTGGCTCAGGATTGCCCATTTTTAATTCCAGGGTTTCTCTGAATTTGAAAGTTATCACTTAGTAAGTTTCCATACCAAGGCTCAATCTAATTAAGTCCGTAGCGTCTACCGATTTCGACA |
| 72 | 6-17 | *Smilax glabra* | TCCATTGAGTCTCTGCACCTATCCCTTTTTGATTCTAGTTTTATAAATTAAACCCCTATTTTATCAAAATAAAGATTTGGCTCAGGATTGCCCATTTTGAATTCCGGGGTTTCTCTGAATTTGGAAGTTACCACTTAGCAGGTTTCCATACCAAGGCTCAAGCTAATCAAGTCCGTAGCGTCTACCGATTTCGA |
| 73 | 6-19 | *Dumasia truncata* | TAATACTCTCCATTGAGTCTCTGCACCTATCCTTTTTTTTGTTTTCGGAAAACGGGATTTGGCTCAGGATTGCCCATTGTGAATTCCAGGGTTTCTCTGAATTTGAAAGTTCTCACTTAGTAAGTTTCCATACCAAGACTCAATCCAATTAAGTCCGTAGCGTCTACCGATTTCGA |
| 74 | 6-20 | *Rhododendron simsii* | TCCATTGAGTCTCTGCACCTATCCTTTTTTTTAGCTTTCGAAATCTTTGTTTGTTTGCGAAAAAAGGATTTGGCTCAGGATTGCCCATTTTTTTTTCCGGGGTTTCTCTGAATTTGAAAGTTATCACTTAGTAGGTTTCCATACCAAGGCTCAATCCAATTAAGTCCGTAGCGTCTACCGATTTCGA |
| 75 | 6-21 | *Lindera aggregata* | TATTCCATTGAGTCTCTGCACCTATCCTTTTTTTGGTTCTCGCTTTCTGAACCCTTGTTTTCTGAAAACAGGATTTGGCTCAGGATTGCCCATTTTTAATTCCAGGGTTTCTCTGAATTTGGAAGTTATCACTTAGTAGGTTTCCATACCAAGGCTCAATCCAACCAAGTCCGTAGCGTCTACCGATTTCGA |
| 76 | 6-22 | *Citrus maxima* | TCCATTGAGTCTCTGCACCTATCCCCCTTTTTCGCTTTCTGAACCCCTGTTTGTTCTTGGAAAAGAGAAGAGGATTTGGCTCAGGATTACCCATTTTTAATTCCTGGGTTTCTCTGAATTTGAAAGTTATCACTTAGTAAGTTTCCATACTAAGGCTCAATCCAATTAAGTCCGTAGCGTCTACCGATTTCGAGAGG |
| 77 | 6-25 | *Morus alba* | TCCATTGAGTCTCTGCACCTATCCTTTTGTATTATCGCTTTCTGAACCCTTGTTTGTTTTCAGAAAACCGGATTTGGCTCAGGATTGCCCATTTTTAATTCCAGGGTTTCTCTGAATTTGAAAGTTATCACTTGGTAGGTTTCCATACCAAGGCTCAATTCAATTAAGTCCGTAGCGTCTACCGATTTCGA |
| 78 | 6-26 | *Cynodon dactylon* | TCCATTGAGTCTCTGCACCTATCCTTTTCCTTTGGGTTCTGGTTTGAGAACCACTTGTTTTTCAAAAAAGGGATTTGGCTCAGGATTGCCCATTTTTCATTCCAGGGTTTCTCTGAATTTGGAAGTTACCACTTAGCAGGTTTCCATACCAAGGCTCAATACAATCAAGTCCGTAGCGTCTACCGATTTCGAAA |
| 79 | 6-27 | *Rhododendron simsii* | TCCATTGAGTCTCTGCACCTATCCTTTTTTTTAGCTTTCGAAATCTTTGTTTGTTTGCGAAAAAAGGATTTGGCTCAGGATTGCCCATTTTTTTTTCCGGGGTTTCTCTGAATTTGAAAGTTATCACTTAGTAGGTTTCCATACCAAGGCTCAATCCAATTAAGTCCGTAGCGTCTACCGATTTCGA |
| 80 | 6-28 | *Callicarpa bodinieri* | TCCATTGAGTCTCTGCACCTATCCTTTTTTTTCGTTTTTTGAACCTTTGTTTTGAGAAAACAGGATTTGGCTCAGGATTGCCCATTTTTATTAATTCCGGGGTTTCTCTGAATTTGAAAGTTCTCACTTAGTAGGTTTCCATACCAAGGCTCAATCCAATTAAGTCCGTAGCGTCTACCGATTTCGA |
| 81 | 6-29 | *Ophiopogon bodinieri* | TCCATTGAGTCTCTGCACCTATCTTTAATATTAGATAAGAAATAATATTATTTCTTATCTGAAATAAGAAATATTTTATATATTTCTTTTTCTCAAAAAGAAGATTTGGCTCAGGATTGCCCATTTTTAATTCCAGGGTTTCTCTGAATTTGGAAGTTAACACTTAGCAAGTTTCCATACCAAGGCTCAATCCAATGCAAGTCCGTAGCGTCTACCGATTTCGA |
| 82 | 6-3 | *Prunus spinosa* | TCCATTGAGTTTTTGCACCTATCCTTTATCCTTTTTTTTTTTCGTTTTTTAAACCTTTGTTTTGAGAAAACAGGATTTGGCTCAGGATTGCCCATTTTTATTAGTTCCGGGGTTTCTCTGAATTTGAAAGTCATCACTTAGCAAGTTTCCATACCAAGGCTCAATCCAATTAAGTCCGTAGCGTCTACCGATTTCGA |
| 83 | 6-31 | *Typha orientalis* | TCCATTGAGTCTCTGCACCTATCCTTTTTTATTCTAGTTTTATAAACCCTTGTTTTCTCAAAATATAAGGATTTGGCTCAGGATTGCCCATTTTTAATTCCAGGGTTTCTCTGAATTTGGAAGTTAACACTTAGCAGGTTTCCATACCAAGGCTCAATCCAATCAAGTCCGTAGCGTCTACCGATTTCGA |
| 84 | 6-34 | *Ampelopsis humulifolia* | TCCATTGAGTCTCTGCACCTATCCTTTTTTTATTATCGTTTTCTGAACCCTTGGTTGTTTTCGGAAAACAGGATTTGGCTCAGGATTGCCCATTTTTTATTCCAGGGTTTCCCTGAATTTGAAAGTTATCACTTAGTAGGTTTCCATATTAAGGCTCAATCCAATTAAGTCCGTAGCGTCTACCGATTTCGA |
| 85 | 6-36 | *Persicaria perfoliata* | TCCATTGAGTCTCTGCACCTATCCTCTTTTTCTTTCCTTTTGGAAAGCAGGAGTTGGCTCAGGATTGCCCATTTCTTTAATTCCAGGGTTTCTCTGAATTTGAAAGTTATCACTTAGTAAGTTTCCATACTAAGGCTCAAACCAATTAAGTCCGTAGCGTCTACCGATTTCGA |
| 86 | 6-37 | *Vitis bryoniifolia* | TCCATTGAGTCTCTGCACCTATCCTTTTTTTATTATCGTTTTCTGAACCCTTGGTTGTTTTCGGAAAACAGGATTTGGCTCAGGATTGCCCATTTTTTATTCCAGGGTTTCCCTGAATTTGAAAGTTATCACTTAGTAGGTTTCCATATTAAGGCTCAATCCAATTAAGTCCGTAGCGTCTACCGATTTCG |
| 87 | 6-38 | *Citrus trifoliata* | TCCATTGAGTCTCTGCACCTATCCCCCTTTTTCGCTTTCTGAACCCCTGTTTGTTCTTGGAAAAGAGAAGAGGATTTGGCTCAGGATTACCCATTTTTAATTCCTGGGTTTCTCTGAATTTGAAAGTTATCACTTAGTAAGTTTCCATACTAAGGCTCAATCCAATTAAGTCCGTAGCGTCTACCGATTTCGA |
| 88 | 6-4 | *Eurya nitida* | CCATTGAGTCTCTGCACCTATCCTTTTTTGATTTTCGCTTTCTGAATCTTTGTTTGTTTTCGAAAAACAGGATTTGGCTCAGGATTGCCCATTTTTATTAATTCCAGGGTTTCTCTGAATTTGAAAGTTATCACTTAGTAGGTTTCCATACCAAGGCTCAATCCAATTAAGTCCGTAGCGTCTACCGATTTCGA |
| 89 | 6-40 | *Michelia maudiae* | TCCATTGAGTCTCTGCACCTATCCTTTTTTGATTCTCGCTTTCTGAACCCTTGTTTTTTGAACACAGGATTTGGCTCAGGATTGCCCATTTTTAATTCCAGGGTTTCTCTGAATTTGGAAGTTACCACTTAGTAGGTTTCCATACCAAGGCTCAATCCAATCAAGTCCGTAGCGTCTACCGATTTCGA |
| 90 | 6-41 | *Musa basjoo* | TCCATTGAGTCTCTGCACCTATCCTTTTAAATTCTAGTTTTATAAACCTTTGTTTTCTCAAAATAAGGATTTGGCTCAGGATTGCCCATTTTTAATTCCAGGGTTTCTCTGAATTTGGAAGTTACCACTTAGCAGGTTTCCATACTAAGGCTCAATCCAATCAAGTCCGTAGCGTCTACCGATTTCGA |
| 91 | 6-5 | *Citrus reticulata* | TCCATTGAGTCTCTGCACCTATCCCCCTTTTTCGCTTTCTGAACCCCTGTTTGTTCTTGGAAAAGAGAAGAGGATTTGGCTCAGGATTACCCATTTTTAATTCCTGGGTTTCTCTGAATTTGAAAGTTATCACTTAGTAAGTTTCCATACTAAGGCTCAATCCAATTAAGTCCGTAGCGTCTACCGATTTCGA |
| 92 | 6-6 | *Lonicera japonica* | TCCATTGAGTCTCTGCACCTATCCTTTTTGATTTTCGCTTTCTGAACCGTTGTTTGTTTTCGGAAAACGTGATTTGGCTCAGGATTGCCCATTTTTATTAATTCCAGGGTTTCTCTGAATTTGAAAGTTATCACTTAGTAAGTTTCCATACCAAGGCTCAATCCAATTAAGTCCGTAGCGTCTACCGATTTCGA |
| 93 | 6-8 | *Symplocos sumuntia* | CCATTGAGTCTCTGCACCTATCCTTTTTTTATTTTCGCTTTCTGAATCTTTTTTTGTTTTCTAAAAACAGGATTTGGCTCAGGATTGCCCATTTTTATTAATTCCAGGGTTTCTCTGAATTTGAAAGTTATCACTTAGTAGGTTTCCATACCAAGGCTCAATACAATTAAGTCCGTAGCGTCTACCGATTTCGA |
| 94 | 6-9 | *Viburnum dilatatum* | TCCATTGAGTCTTTGCACCTATCCTTTTTTTTTCGCTTCTTTGTTTGTTTTCGGAAAACAGGATTTGGCTCAGGATTGCCCATTTTATTAATTCCAGGGTTTCTCTGAATTTGAAAGTTCTCACTTAGTAGGTTTCCATACCAAGGCTCAATTCAATTAAGTCCGTAGCGTCTACCGATTTCGA |
| 95 | 7-11 | *Cyperus rotundus* | TCCATTGAGTCTCTGCACCTATCTTTAATATTAGACTAAGAAAAAATATTATTTCTTATCTGAAATAAGAAATATTTTATATATTTCTTTTTCTCAAAAAGAAGATTTGGCTCAGGATTGCCCATTTTTAATTCCAGGGTTTCTCTGAATTTGGAAGTTAACACTTAGCAAGTTTCCATACCAAGGCTCAATCCAATGCAAGTCCGTAGCGTCTACCGATTTC |
| 96 | 7-12 | *Veronica polita* | TCCATTGAGTCTCTGCACCTATCCCCTTTTCACTTCCTGAACTAGAAGACAGGATTTGGCTCAGGATTGCCCATTTTTATTAATTCCGGGGTTTCTCTGAATTTGAAAGTTATCACTTAGTAGGTTTCCATACCAAGGCTCAATCTAATTAAGTCCGTAGCGTCTACCGATTTCGA |
| 97 | 7-16 | *Salvia plebeia* | TCCATTGAGTCTCTGCACCTATCCTTTTTTTTGAGAAAAAAGGATTTGGCTCAGGATTGCCCATTTTTATTAATTCCGGGGTTTCTCTGAATTTGAAAGTTATCACTTAGTAAGTTTCCATACCAAGGCTCAATCTAATTAAGTCCGTAGCGTCTACCGATTTCGA |
| 98 | 7-17 | *Lonicera japonica** | TCCATTGAGTCTCTGCACCTATCCTTTTTGATTTTTGCTTTCTAAACCCCTGTTTTCGGAAAACCGGATTTGGCTCAGGATTGCCCATTTTTATTAATTCCGGGGTTTCTCTGAATTTGAAAGTTTTCACTTAGTAGGTTTCCATACCAAGGCTCAATCCAATTAAGTCCGTAGCGTCTACCGATTTCGA |
| 99 | 7-2 | *Bambusa multiplex* | TCCATTGAGTCTCTGCACCTATCCTTTTTTTATTCTAGTTTTATAAACCCTTGTTTTCTCAAAATAAAGATTTGGCTCAGGATTGCCCATTTTAAATTCCAGGGTTTCTCTGAATTTGGAAGTTACCACTTAGTAGGTTTCCATACCAAGGCTCAATCCAATCAAGTCCGTAGCGTCTACCGATTTCGA |
| 100 | 7-20 | *Trichosanthes kirilowii** | TCCATTGAGTCTCTGCACCTATCCTTTTTTTTTCGGAAAAAGGATTTGGCTCAGGATTGCCCATTTTTAATTCCAGGGTTTCTCTGAATTTGAAAGTTATCACTTAGTAGGTTTCCATACCAAGGCTCAATCCAATTAAGTCCGTAGCGTCTACCGATTTCGA |
| 101 | 7-21 | *Trichosanthes kirilowii** | TCCATTGAGTCTCTGCACCTATCCTTTTTTTTTCGGAAAAAGGATTTGGCTCAGGATTGCCCATTTTTAATTCCAGGGTTTCTCTGAATTTGAAAGTTATCACTTAGTAGGTTTCCATACCAAGGCTCAATCCAATTAAGTCCGTAGCGTCTACCGATTTCGA |
| 102 | 7-22 | *Biancaea decapetala* | CCATTGAGTCTCTGCACCTATCCTTTTTTTATTCTCCCTTTCTGAACTCTTCTTGGTTTTCGGAAAACAGGATTTGGCTCAGGATTGCCCATTGTTAATTCCAGGGTTTCTCTGAATTTGAAAGTTCTCACTTGGTATATTTCCATACCAAGGCTCAATCCAATTAAGTCCGTAGCGTCTACCGATTTCG |
| 103 | 7-27 | *Fargesia spathacea* | TCCATTGAGTCTCTGCACCTATCCTTTTCCTTTGGATTCTAGTTCGAGAACCACTTGTTTTCTCAAAACACGGATTTGGCTCAGGATTGCCCTTTTTTAATTCCAGGGTTTCTCTGAATTTGGAAGTTACCACTTAGCAGGTTTCCATACCAAGGCTCAATACAATCAAGTCCGTAGCGTCTACCGATTTCGA |
| 104 | 7-28 | *Gossypium hirsutum* | CCATTGAGTCTCTGCACCTATCCTTTCCTTTTTTTCTTATTCTCGCTTGCTGAACCTTTGTTCATGTTTATTTTCGTAAAATAATAAAATAATAGGATTTGGCTCAGGATTGCCCATTTTTCATTCCAGGGTTTCTCTGAATTTGAAAGTTATCACTTAGTAGGTTTCCATACCAAGGCTCAATCCAATTAAGTCCGTAGCGTCTACCGATTTCGA |
| 105 | 7-29 | *Sambucus javanica* | TCCATTGAGTCTCTGCACCTATCCTTTTTTATTTTCGCTTTCTGAACCTTTGTTTGTTTTCGGAAAACAGGATTTGGCTCAGGATTGCCCATTTTTATTAATTCCAGGGTTTCTCTGAATTTGAAAGTTCTCACTTAGTAGGTTTCCATACCAAGGCTCAATCCAATTAAGTCCGTAGCGTCTACCGATTTCG |
| 106 | 7-3 | *Juncus effusus* | TCCATTGAGTCTCTGCACCTATCTTTTTTGATTTGAGTTTTTCTATAAAAACCAATTTTATCAAAATAAAGATTTGGCTCAGGATTGCCCATTTTGAATTCCAGGGTTCCTCTGAATTTGGAAGTTACCACTTAGCAAGTTTCCATACCAAGGCTCAATCCAATCAAGTCCGTAGCGTCTACCGATTTCG |
| 107 | 7-30 | *Setaria plicata* | TCCATTGAGTCTCTGCACCTATCCTTTTCCTTTGGGTTCTAGTTTGAGAACCACTTGTTTTGTTTTTTCAAAAAAGGGATTTGGCTCAGGATTGCCCATTTTTCATTCCAGGGTTTCTCTGAATTTGGAAGTTATCACTTAGCAGGTTTCCATACCAAGGCTCAATACAATCAAGTCCGTAGCGTCTACCGATTTCG |
| 108 | 7-32 | *Buddleja lindleyana* | TCCATTGAGTCTCTGCACCTATCCTTTTTTCGCCTTCTGAACCTTTGTTTTGAGACAACAGGATTTGGCTCAGGATTGCCCATTTTTATTAATTCCGGGGTTTCTCTGAATTTGAAAGTTATCACTTAGTAGGTTTCCATACCAAGGCTCAATCCAATTAAGTCCGTAGCGTCTACCGATTTCGA |
| 109 | 7-33 | *Reynoutria japonica* | TCCATTGAGTCTCTGCACCTATCCCTTTTTCTTCCTTTTGGAAAGAAGGAGTTGGCTCAGGATTGCCCATTTTTTTAATTCCAGGGTTTCTCTGAATTTGAAAGTTCTCACTTAGTAGGTTTCCATACTAAGGCTCAAACCAATTAAGTCCGTAGCGTCTACCGATTTCGA |
| 110 | 7-34 | *Trema cannabina* | CCATTGAGTCTCTGCACCTATCCTATTCTTTTTTATTATCGCTTTCGGAATCCTTGTTTGTTTTCAGAAAACCGGATTTGGCTCAGGATTACCCCATTTTTTTAATTCCGGGGTTTCTCTGAATTTGAAAGTTATCACTTGGTAGGTTTCCATACCAAGGCTCAATCCAATTAAGTCCGTAGCGTCTACCGATTTC |
| 111 | 7-36 | Physalis philadelphica | TCCATTGAGTCTCTGCACCTATCCTTTTTTTCTGAACCTTTGTTTGTTTTCAGAAAACAGGATTTGGCTCAGGATTGCCCATTTTTGTTAATTCCAGGGTTTCTCTGAATTTGAAAGTGATCACTTAGTAAGTTTCCATACCAAGGCTCAATCCAATTAAGTCCGTAGCGTCTACCGATTTCGA |
| 112 | 7-40 | *Houttuynia cordata* | TCCATTGAGTCTCTGCACCTATCCTTTTTTTGATTTGATTTTCGCTTTCTGAACTCTTTTTTTTTATGAAAATAGGATTTGGCTCAGGATTGCCCATTTTTCATTCCAGGGTTTCTCTGAATTTGGAAGTTACCACTTAGTAGGTTTCCATACCAAGGCTCAATCCAATCAAGTCCGTAGCGTCTACCGATTTCGA |
| 113 | 7-44 | *Catharanthus roseus* | TCCATTGAGTCTCTGCACCTATCCCTTTTTCGTTTTCTGAACCTTTGTTTGTGTTTGTGGAAAACTGGATTTGGCTCAGGATTGCCCCTTTTATTAATTCCGGGGTTTCTCTGAATTTGAAAGTTATCACTTAGTAGGTTTCCATACCAAGGCTCAATCCAATTAAGTCCGTAGCGTCTACCGATTTCGA |
| 114 | 7-48 | *Ligustrum sinense* | TCCATTGAGTCTCTGCACCTATCCTTTTTTCTTTCTGAACCTTTATTTTGGGAAAACAGGATTTGGCTCAGGATTGCCCATTTTTATTAATTCCGGGGTTTCTCTGAATTTGAAAGTTACCACTTAGTAGGTTTCCATACCAAGGCTCAATCCAATTAAGTCCGTAGCGTCTACCGATTTCG |
| 115 | 7-52 | *Celosia cristata* | TCCATTGAGTCTCTGCACCTATCCTTTGTTTTTTATTCGTGCTTTCTGAATCCTTCTTTTTTTACTTTTCAAAAAAGGAGTTGGCTCAGGATTGCCCATTTTTATTAATTCCAGGGTTTCTCTGAATTTGAAAGTTTTCACTTAGTAGGTCTCCATACTAAGGCTCAAGCCAATTAAGTCCGTAGCGTCTACCGATTTCGA |
| 116 | 7-6 | *Nerium oleander* | TCCATTGAGTCTCTGCACCTATCCTTGTTTTCGTTTTTTGAACCTTTGTTTGTGGAAAATAGGATTTGGCTCAGGATTGCCCTTTTTATTAATTCCGGGGTTTCTCTGAATTTGAAAGTCATCACTTAGTAGGTTTCCTTACCAAGGCTCAATCCAATTAAGTCCGTAGCGTCTACCGATTTCG |
| 117 | 7-66 | *Celtis biondii* | TCCATTGAGTCTCTGCACCTATCCGATTCTTTTTTATTATCGCTTTCTGAATCCTTGTTTGTTTTCAGAAAACCGGATTTGGCTCAGGATTGCCCATTTTTTTTAATTCCAGGGTTTCTCTGAATTTGAAAGTTATCACTTGGTAGGTTTCCATACCAAGGCTCAATCCAATTAAGTCCGTAGCGTCTACCGATTTCGA |
| 118 | 7-67 | *Photinia prunifolia* | TCCATTGAGTCTCTGCACCTATCCTTTTTTATTTTCGGTTTATGAAACCCTTGTTTATTTTCATAAAACAGGATTTGGCTCAGGATTGCCCATTTTTAATTCCAGGGTTTCTCTGAATTTGAAAGTTCTCACTTGGTAGGTTTCCATACCAAGGCTCAATCCAATTAAGTCCGTAGCGTCTACCGATTTCGA |
| 119 | 7-68 | *Schnabelia terniflora* | CATCTCCATTGAGTCTCTGCACCTATCCTTTGTTTTGAAAAAACAGGATTTGGATCAGGATCGCCCATTTTTATTAATTCCGGGGTTTCTCTGAATTTGAAAGTTATCACTTAGTAGGTTTCCCACACTAAGGCTCAATTTAATTATAATTAAGTCCGTAGCGTCTACCGATTTCGT |
| 120 | 8-1 | *Agrimonia pilosa* | CCATTGAGTCTCTGCACCTATCCTTTATTTTTTCTCGCTTTGTGAAACCCTTGTTTGTTTTCATAAAACGGGATTTGGCTCAGGATTGCCCATTTTTAATTCCAGGGTTTCTCTGAATTTGAAAGTTATCACTTGGTAGGTTTCCATACCAAGGCTCAATTCAATTTAAGTCCGTAGCGTCTACCGATTTCG |
| 121 | 8-11 | *Euonymus alatus* | TCCATTGAGTCTCTGCACCTATCCTTGTGTTATTCTTGCTTTCTGAATCTTTCTTTGTTTTCGGACAATAGGATTTGGCTCAGGATTGCCCATTTTTAATTCCAGGGTTTCTCTGAATTTGAAAGTTATCACTTAGTAAGTTTCCGTACCAAGGCTCAATCCAATTAAGTCCGTAGCGTCTACCGATTTCG |
| 122 | 8-12 | *Ardisia crenata* | TCCATTGAGTCTCTGCACCTATCCCTCTTTTTTATTTTCCTTTAATCTTTGTTTTCGAAAAAGAGGATTTGGCTCAGGATTGCCCATTTTTATTAATTCCAGGGTTTCTCTGAATTTGAAAGTTCTCACTTAGTAGGTTTCCATACTAAGGCTCAATCCAATTAAGTCCGTAGCGTCTACCGATTTCGA |
| 123 | 8-14 | *Pilea notata* | TCCATTGAGTCTCTGCACCTATCCTATCTTTTTTATTTTCGCTTTCTGAACGCTTGTTTGTTTTGATAAAACCGGATTTGGCTCAGGATTGCCCATTTTTACTTCCAGGGTTTCTCTGAATTTGAAAGTTATCACTCGGTAGGTTTCCATACCAAGGCTCAGTCCAATTAAGTCCGTAGCGTCTACCGATTTCGA |
| 124 | 8-15 | *Ficus pumila** | TCCATTGAGTCTCTGCACCTATCCTTTTTTATTATCGCCTTCTGAACCCTTGTTTGTTTTCAGAAAACCGGATTTGGCTCAGGATTGCCCATTTTTAATTCCAGGGTTTCTCTGAATTTGAAAGTTATCACTTGGTAGGTTTCCATACCAACGCTCAATTCAATTCAATTAAGTCCGTAGCGTCTACCGATTTCGAGACG |
| 125 | 8-17 | *Akebia trifoliata* | TCCATTGAGTCTCTGCACCTATCCTTCCTTTATTTTTTTAATCTCGCTTTCTGAACCTTTTTTTTTCTGAAAACAGGATTTGGCTCAGGATTGCCCATTTTTCATTCCAGGGTTTCTCTGAATTTGAAAGTTATCACTTAGTAGGTTTCCATACCAAGGCTCAATCCAATCAAGTCCGTAGCGTCTACCGATTTCGACGGG |
| 126 | 8-18 | *Kadsura longipedunculata* | TCCATTGAGTCTCTGCACCTATCCCTACCTTTTTTTTATTCTCGCTTTCTAAACTTCTAAACCATTGTTTTCTGAAAACAGGATTTGGCTCAGGATTACCCATTTTTAATTCCAGGGTTTCTCTGAATTTGGAAGCTACCACTTAGTAGGTTTCCATACTAAGGCTCAATCCAATCAAGTCCGTAGCGTCTACCGATTTCGA |
| 127 | 8-23 | *Euscaphis japonica* | TCCATTGAGTCTCTGCACCTATCCTTTTTGTATTCTCGCTTGCCGAATTCTTCTTTGTTTTCGTAAACCAGGATTTGGCTCAGGATTGCCCATTTTTTATTCCAGGGTTTCTCTGAATTTGAAAGTTATCACTTAGTAGGTTTCCATACCAAGGCTCAATCCAATTAAGTCCGTAGCGTCTACCGATTTCGA |
| 128 | 8-29 | *Akebia trifoliata subsp. australis* | TCCATTGAGTCTCTGCACCTATCCTTCCTTTATTTTTTTAATCTCGCTTTCTGAACCTTTTTTTTTCTGAAAACAGGATTTGGCTCAGGATTGCCCATTTTTCATTCCAGGGTTTCTCTGAATTTGAAAGTTATCACTTAGTAGGTTTCCATACCAAGGCTCAATCCAATCAAGTCCGTAGCGTCTACCGATTTCGA |
| 129 | 8-3 | *Pleuropterus multiflorus** | TCCATTGAGTCTCTGCACCTATCCTTTTTTATTCTAGTTTTATAAACCCTTGTTTTCTCAAAATATAAGGATTTGGCTCAGGATTGCCCATTTTTAATTCCAGGGTTTCTCTGAATTTGGAAGTTAACACTTAGCAGGTTTCCATACCAAGGCTCAATCCAATCAAGTCCGTAGCGTCTACCGATTTCGA |
| 130 | 8-31 | *Cocculus orbiculatus* | TCCATTGAGTCTCTGCACCTATCCATTTTTGAGTCTCGCTTTCTGAACTCTTTTTTGTTTTCTGAAAACAGGATTTGGCTCAGGATTGCCCATTTTTAATTCCAGGGTTTCTCTGAATTTGAAAGTTTTCACTTAGTAGGTTTCCATACCAAGGCTCAATCCAATCAAGTCCGTAGCGTCTACCGATTTCGA |
| 131 | 8-32 | *Dioscorea polystachya* | TCCATTGAGTCTCTGCACCTATCCTTTTTGATTTTTGATTCTAGTTTGTTTTGTTTTTTTATAAACCAAAGGGTTTATAAAAAACAAAACAAATAAAGATTTGGCTCAGGATTGCCCATTTCAAATTCCAGGGTTTCTCTGAATTTGGAAGTTATCACTTAGCAGGTTTCCATACCAAGGCTCAATTCAATCAAGTCCGTAGCGTCTACCGATTTCGA |
| 132 | 8-33 | *Tubocapsicum anomalum* | TCCATTGAGTCTCTGCACCTATCCTTTTTTTCTGAACCTTTGTTTGTTTTCAGAAAACAGGATTTGGCTCAGGATTGCCCATTTTTGTTAATTCCAGGGTTTCTCTGAATTTGAAAGTGATCACTTAGTAAGTTTCCATACCAAGGCTCAATCCAATTAAGTCCGTAGCGTCTACCGATTTCGA |
| 133 | 8-34 | *Salvia chinensis* | TCCATTGAGTCTCTGCACCTATCCTTTTTTTTGAAAAAACAGGATTTGGCTCAGGATTGCCCATTTTTATTAATTCCGGGGTTTCTCTGAATTTGAAAGTTATCACTTAGTAAGTTTCCATACCAAGGCTCAATCTAATTAAGTCCGTAGCGTCTACCGATTTCGA |
| 134 | 8-36 | *Litsea cubeba* | TCCATTGAGTCTCTGCACCTATCCTTTTTTTGGTTCTCGCTTTCTGAACCCTTGTTTTCTGAAAACAGGATTTGGCTCAGGATTGCCCATTTTTAATTCCAGGGTTTCTCTGAATTTGGAAGTTATCACTTAGTAGGTTTCCATACCAAGGCTCAATCCAACCAAGTCCGTAGCGTCTACCGATTTCGAAAT |
| 135 | 8-37 | *Tripterospermum chinense* | TCCATTGAGTCTCTGCACCTATCCTTTTTCTTTCTGAGCTTTTCTTTTTGCTTTTTCAAAAAAAAGGATTTGGCTCAGGATTACCCTTTATTATTATTATATTATTATTAAATTATTAATTCCAGGGTTTCTCTGAATTTGAAAATTATCACTTAGTAAGTTTCCATACCAAGGCTCAATCCAATTAAGTCCGTAGCGTCTACCGATTTCGA |
| 136 | 8-38 | *Smilax glaucochina* | AGTTTCCATTGAGTCTCTGCACCTATCCCTTTTTGATTCTAGTTTTATAAATTAAACCCCTATTTTATCAAAATAAAGATTTGGCTCAGGATTGCCCATTTTGAATTCCGGGGTTTCTCTGAATTTGGAAGTTACCACTTAGCAGGTTTCCATACCAAGGCTCAAGCTAATCAAGTCCGTAGCGTCTACCGATTTCG |
| 137 | 8-4 | *Lysimachia patungensis* | TCCATTGAGTCTCTGCACCTATCCCCCTTTTTTATTTTCCTTTAAATCTTTGTTTTCGAAAAAGAGGATTTGGCTCAGGATTGCCCATTTTTATTAATTCCAGGGGTTCTCTGAATTTGAAAGTTCTCACTTAGCAGGTTTCCATACTAAGGCTCAATGCAATTAAGTCCGTAGCGTCTACCGATTTCGA |
| 138 | 8-41 | *Hypericum monogynum* | TCCATTGAGTCTCTGCACCTATCCTTTTTTTTCTGTTTATGAATCTTTCTTTGTTTTCGGAAAGCCGGATTTGGCTCAGGATTGCCCATTTTTTATTCCAGGGTTTCTCTGAATTTGAAAGTTATCACTTGCTAAGTTTCCATACCAAGGCTCAATCCAATTAAGTCCGTAGCGTCTACCGATTTCGA |
| 139 | 8-46 | *Ardisia escallonioides* | TCCATTGAGTCTCTGCACCTATCCCTCTTTTTTATTTTCCTTTAATCTTTGTTTTCGAAAAAGAGGATTTGGCTCAGGATTGCCCATTTTTATTAATTCCAGGGTTTCTCTGAATTTGAAAGTTCTCACTTAGTAGGTTTCCATACTAAGGCTCAATCCAATTAAGTCCGTAGCGTCTACCGATTTCGA |
| 140 | 8-50 | *Corydalis pallida* | TGTGGTCCATTGAGTCTCTGCACCTATCCTTTTTGGATTCTTGTTTTCGGAACCCCCCCCCTTTTTTCTGAAAACAGGATTTGGCTCAGGATTGCCCATTTTTAATTCCAGGGTTTCTCTGAATTTGAAAGTTATCACTTAGTAGGTTTCCATACCAAGGCTCAATCCAATCAAGTCCGTAGCGTCTACCGATTTCGACGCG |
| 141 | 8-51 | *Zanthoxylum armatum* | CCATTGAGTCTCTGCACCTATCTCCCTTTTTCACTTTCTGAACCCTTGTTTGTTCTTGGAAAAGAAGATTTGGCTCAGGATTGCCCATTTTTAATTCCTGGGTTTCTCTGAATTTGAAAGTTATCACTTAGTAGGTTTCTATACTAAGGCTCAATCCAATTAAGTCCGTAGCGTCTACCGATTTCGA |
| 142 | 8-52 | *Salvia miltiorrhiza* | TCCATTGAGTCTCTGCACCTATCCTTTTTTTTGAAAAAACAGGATTTGGCTCAGGATTGCCCATTTTTATTAATTCCGGGGTTTCTCTGAATTTGAAAGTTATCACTTAGTAAGTTTCCATACCAAGGCTCAATCTAATTAAGTCCGTAGCGTCTACCGATTTCGACCC |
| 143 | 8-54 | *Alternanthera philoxeroides* | TCCATTGAGTCTCTGCCCCTATCCTTTGTTTTTTTTTTATTCTTGTTTTCTGAATTCTTATTTTTTCCTAATACTAATAAAAGGAGTTGGCTCAGGATTGCCCCTTTTTTTTAATTCCAGGGTTTCTCTGAATTTGAAAGTTTTCACTTAGTAGGTCTCCATACTAAGGCTCAAGCCAATTAAGTCCGTAGCGTCTACCGATTTCGA |
| 144 | 8-56 | *Mucuna sempervirens* | TCCATTGAGTCTCTGCACCTATCCTTTTTTTTATTATCACTTTCGGAACTTTTCTTTGTTTTCGAAAAACAGGATTTGGCTCAGGATTGCCCATTGTGAATTCCAGGGTTTCTCTGAATTTGAAAGTTCTCACTTGGTAAGTTTCCATACCAAGACTCAATCCAATTAAGTCCGTAGCGTCTACCGATTTCGA |
| 145 | 8-58 | *Rhynchosia volubilis* | TCCATTGAGTCTCTGCACCTATCCCTTTTTTATTATCACTTTCTGAACTTTTCTTTATTTTCGGAAAACAGGATTTGGCTCAGGATTGCCCATTGTGAATTCCAGGGTTTCTCTGAATTTGAAAGTTCTCACTTGGTAAGTTTCCATACCAAGACTCAATCCAATTAAGTCCGTAGCGTCTACCGATTTCGA |
| 146 | 8-6 | *Ardisia japonica* | TCCATTGAGTCTCTGCACCTATCCCTCTTTTTTATTTTCCTTTAATCTTTGTTTTCGAAAAAGAGGATTTGGCTCAGGATTGCCCATTTTTATTAATTCCAGGGTTTCTCTGAATTTGAAAGTTCTCACTTAGTAGGTTTCCATACTAAGGCTCAATCCAATTAAGTCCGTAGCGTCTACCGATTTCGA |
| 147 | 8-61 | *Symplocos stellaris* | TCCATTGAGTCTCTGCACCTATCCTTTTTTTATTTTCGCTTTCTGAATCTTTTTTTGTTTTCTAAAAACAGGATTTGGCTCAGGATTGCCCATTTTTATTAATTCCAGGGTTTCTCTGAATTTGAAAGTTATCACTTAGTAGGTTTCCATACCAAGGCTCAATACAATTAAGTCCGTAGCGTCTACCGATTTCGAGACC |
| 148 | 8-62 | *Potentilla kleiniana* | TCCATTGAGTCTCTGCACCTATCCTTTATTTATTCTCGCTTTATGAAACCCTTGTTTGTTTTCATAAAACGGGATTTGGCTCAGGATTGCCCATTTTTAATTCCAGGGTTTCTCTGAATTTGAAAGTTATCACTTGGTAGGTTTCCATACCAAGGCTCAATCCAATTAAGTCCGTAGCGTCTACCGATTTCGAAACT |
| 149 | 8-64 | *Ampelopsis glandulosa** | TCCATTGAGTCTCTGCACCTATCCTTTTTTTATTATCGTTTTCTGAACCCTTGGTTGTTTTCGGAAAACAGGATTTGGCTCAGGATTGCCCATTTTTTATTCCAGGGTTTCCCTGAATTTGAAAGTTATCACTTAGTAGGTTTCCATATTAAGGCTCAATCCAATTAAGTCCGTAGCGTCTACCGATTTCGA |
| 150 | 8-7 | *Rubus hirsutus* | TCCATTGAGTCTCTGCACCTATCCTTTATTTATTCTCGCTTTCTGAAACCCTTGTTTGTTTTCATAAAACGGGATTTGGCTCAGGATTGCCCATTTTTAATTCCAGGGTTTCTCTGAATTTGAAAGTTATCACTTGGTAGGTTTCCATACCAAGGCTCAATCCAATTAAGTCCGTAGCGTCTACCGATTTCGAAAGC |
| 151 | 8-70 | *Trigonotis peduncularis* | TCCATTGAGTCTCTGCACCTATCCTTTTTTCTTTTTCAACTTTTGTTTGTTTTCGGAAAACCGGATTTGGCTCAGGATTGCCCATTTTTATTAATTCCGGGGTTTCTCTGAATTTGAAAGTTGTCACTTAGTAGGTTTCCATACCAAGGCTCAATCCAATTAAGTCCGTAGCGTCTACCGATTTCGA |
| 152 | 8-72 | *Hedyotis chrysotricha* | TCCATTGAGTCTCTGCACCTATCCTTTATTTCGTTTTTTGAACCTTTGTTTTGAAAAAACAGGATTTGGATCAGGATCGCCCATTTTTCTTAATTCCGGGGTTTCTCTGAATTTGAAAGTTATCACTTAGTAGGTTTCCCACACTAAGGCTCAATTTAATTATAATTAAGTCCGTAGCGTCTACCGATTTCGACAC |
| 153 | 8-8 | *Persicaria criopolitana* | TCCATTGAGTCTCTGCACCTATCCCTTTTTTCTTTCCTTTTTGAAAGCAGGAGTTGGCTCAGGATTGCCCATTTTTTTAATTCCAGGGTTTCTCTGAATTTGAAAGTTATCACTTAGTAAGTTTCCATACTAAGGCTCAAACCAATTAAGTCCGTAGCGTCTACCGATTTCGA |
| 154 | 8-9 | *Ficus sarmentosa var. impressa* | TCCATTGAGTCTCTGCACCTATCCTTTTTTATTATCGCCTTCTGAACCCTTGTTTGTTTTCAGAAAACCGGATTTGGCTCAGGATTGCCCATTTTTAATTCCAGGGTTTCTCTGAATTTGAAAGTTATCACTTGGTAGGTTTCCATACCAACGCTCAATTCAATTCAATTAAGTCCGTAGCGTCTACCGATTTCGA |
| 155 | 9-1 | *Ligustrum vulgare* | TCCATTGAGTCTCTGCACCTATCCTTTTTTCTTTCTGAACCTTTATTTTGGGAAAACAGGATTTGGCTCAGGATTGCCCATTTTTATTAATTCCGGGGTTTCTCTGAATTTGAAAGTTACCACTTAGTAGGTTTCCATACCAAGGCTCAATCCAATTAAGTCCGTAGCGTCTACCGATTTCGA |
| 156 | 9-11 | *Urena lobata* | TCCATTCTGTCTCTGCACCTATCCTTTCCTTTTTTTATTATTCTCGCTTGCTGAACTTTTGTTTCTGTTTATTTTCGTAAAATAATAGGATTTGGCTCAGGATTGCCCATTTTTCATTCCAGGGTTTCTCTGAATTTGAAAGTTATCACTTAGTAGGTTTCCATACCAAGGCTCAATCCAATTAAGTCCGTAGCGTCTACCGATTTCGA |
| 157 | 9-13 | *Pyracantha fortuneana* | TCCATTGAGTCTCTGCACCTATCCTTTATTTATTCTCGCTTTCTGAAACCTTGTTTGTTTTCATAAAACGGGATTTGGCTCAGGATTGCCCATTTTTAATTCCAGGGTTTCTCTGAATTTGAAAGTTATCACTTGGTAGGTTTCCATACCAAGGCTCAATCCAATTAAGTCCGTAGCGTCTACCGATTTCGA |
| 158 | 9-17 | *Deutzia crenata* | TCCATTGAGTCTCTGCACCTATCCTTTTTTTATTTTCATTTTCTGAACTTTTGTTTGTTTTCGGAAAACAGGATTTGGCTCAGGATTGCCCATTTTTATTAATTCCAGGGTTTCTCTGAATTTGAAAGTTATCACTTAGTAGGTTTCCATACCAAGGCTCAATCCAATTAAGTCCGTAGCGTCTACCGATTTCGA |
| 159 | 9-19 | *Glycine max* | TCCATTGAGTCTCTGCACCTATCCCTTTTTTATTATCACTTTATGAACTTTTCTTTGTTTTCGGAAAACAGGATTTGGCTCAGGATTGCCCTTTGTGAATTCCAGGGTTTCTCTGAATTTGAAAGTTCTCACTTGGTAAGTTTCCATACCAAGACTCAATCCAATTAAGTCCGTAGCGTCTACCGATTTCGAAACG |
| 160 | 9-3 | Pleuropterus multiflorus* | TCCATTGAGTCTCTGCACCTATCCTTTTTTCTTCCTTTTGGAAAGAAGGAGTTGGCTCAGGATTGCCCATTTTTTTAATTCCAGGGTTTCTCTGAATTTGAAAGTTCTCACTTAGTAGGTTTCCATACTAAGGCTCAAACCAATTAAGTCCGTAGCGTCTACCGATTTCGA |
| 161 | 9-6 | *Coriandrum sativum* | TCCATTGAGTCTCTGCACCTATCCCTTTTTCACCTTCTGGGCCTTTGTTTGTTTTTGGAAAATAGGATTTGGCTCAGGATTGCCCATTTTTATTAATTCCGGGGTTTCTCTGAATTTGAAAGTTCTCACTTAGTAGGTTTCCATACCAAGGCTCAATCCAATTAAGTCCGTAGCGTCTACCGATTTCGATTT |
| 162 | 9-9 | *Corydalis incisa* | TCCATTGAGTCTCTGCACCTATCCTTTAGGTGGATTCTTGTTTTCGGAACCCCCCCTTTTTTTTTCTGAAAAATAGGATTTGGCTCAGGATTGCCCATTTTTGATTCCAGGGTTTCTCTGAATTTGAAAGTTGTCACTTAGTAGGTTTCCATACTAAGGCTCAATCCAATCAAGTCCGTAGCGTCTACCGATTTCGA |

Table Note: “*” indicates the collection of duplicate plant samples(6)
